# Functional knockout of the TRPV1 channel has no effect on the exercise pressor reflex in rats

**DOI:** 10.1113/JP285267

**Published:** 2023-10-25

**Authors:** Laura Anselmi, Guillaume P. Ducrocq, Victor Ruiz-Velasco, Sean D. Stocker, Shannon P. Higgins, Marc P. Kaufman

**Affiliations:** 1 Heart and Vascular Institute Penn State College of Medicine, Hershey, PA, USA; 2 Mitochondria, Oxidative Stress and Muscular Protection Laboratory (UR 3072), Faculty of Medicine, University of Strasbourg, Strasbourg, France; 3 Department of Anesthesiology and Perioperative Medicine, Penn State College of Medicine, Hershey, PA, USA; 4 Department of Neurobiology, University of Pittsburgh, Pittsburgh, PA, USA

**Keywords:** autonomic nervous system, capsaicin, metaboreceptors, neural control of the circulation, thin fibre muscle afferents

## Abstract

The role played by the transient receptor potential vanilloid 1 (TRPV1) channel on the thin fibre afferents evoking the exercise pressor reflex is controversial. To shed light on this controversy, we compared the exercise pressor reflex between newly developed TRPV1^+/+^, TRPV1^+/−^ and TRPV1^−/−^ rats. Carotid arterial injection of capsaicin (0.5 *μ*g), evoked significant pressor responses in TRPV1^+/+^ and TRPV1^+/−^ rats, but not in TRPV1^−/−^ rats. In acutely isolated dorsal root ganglion neurons innervating the gastrocnemius muscles, capsaicin evoked inward currents in neurons isolated from TRPV1^+/+^ and TRPV1^+/−^ rats but not in neurons isolated from TRPV1^−/−^ rats. The reflex was evoked by stimulating the tibial nerve in decerebrated rats whose femoral artery was either freely perfused or occluded. We found no difference between the reflex in the three groups of rats regardless of the patency of the femoral artery. For example, the peak pressor responses to contraction in TRPV1^+/+^, TRPV1^+/−^ and TRPV1^−/−^ rats with patent femoral arteries averaged 17.1 ± 7.2, 18.9 ± 12.4 and 18.4 ± 8.6 mmHg, respectively. Stimulation of the tibial nerve after paralysis with pancuronium had no effect on arterial pressure, findings which indicated that the pressor responses to contraction were not caused by electrical stimulation of afferent tibial nerve axons. We also found that expression levels of acid-sensing ion channel 1 and endoperoxide 4 receptor in the L4 and 5 dorsal root ganglia were not upregulated in the TRPV1^−/−^ rats. We conclude that TRPV1 is not needed to evoke the exercise pressor reflex in rats whose contracting muscles have either a patent or an occluded arterial blood supply.

## Graphical Abstract

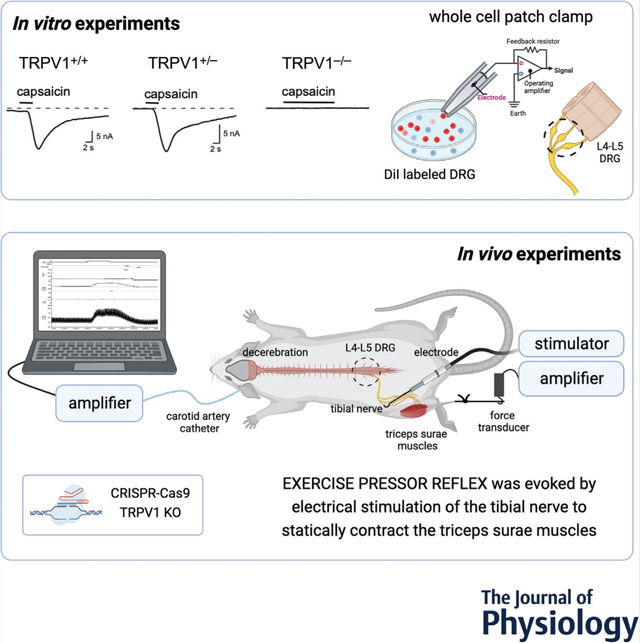

In decerebrated rats, CRISPR-induced functional knockout of TRPV1 channels had no effect on the magnitude of either the exercise pressor reflex evoked by static contraction of the triceps surae muscles or the muscle mechanoreflex evoked by calcaneal tendon stretch. Evidence of functional TRPV1 channel knockout was provided *in vitro* by showing that capsaicin, a TRPV1 agonist, evoked inward currents in TRPV^+/+^ and TRPV1^+/−^ dorsal root ganglion neurons innervating the triceps surae muscles, but did not evoke inward currents in their TRPV1^−/−^ counterparts. Evidence of functional TRPV1 channel knockout *in vivo* was provided by showing that capsaicin, injected into the systemic circulation, evoked pressor responses in TRPV1^+/+^ and TRPV1^+/−^ rats but did not evoke pressor responses in TRPV1^−/−^ rats.

## Introduction

The exercise pressor reflex is evoked by contraction of limb skeletal muscle and is manifested by increases in the sympathetic outflow to the heart and vascular tree, by decreases in vagal outflow to the heart (Gladwell & Coote, 2002; McMahon & McWilliam, 1992), and by increases in diaphragmatic activity (Coote et al., 1971; McCloskey & Mitchell, 1972; Mitchell et al., 1983). In healthy preparations with freely perfused muscles, these effects, in combination with sympatholysis (Fadel et al., 2012; Remensnyder et al., 1962), result in increases in heart rate, cardiac contractility, arterial blood pressure, ventilation and blood flow to the exercising muscles (Amann et al., 2010; O’Leary et al., 1999). The sensory arm of the exercise pressor reflex is comprised of group III and IV muscle afferents (McCloskey & Mitchell, 1972), of which the former are primarily mechanosensitive, and the latter are primarily metabosensitive (Kaufman et al., 1983; Mense & Stahnke, 1983). Because of the respective sensitivities of these thin fibre muscle afferents, the exercise pressor reflex has a mechanical component and a metabolic component.

The specific nature of the channels on the sensory endings of group III and IV afferents that transduce mechanical and metabolic stimuli arising in contracting muscles have been extensively investigated. For example, Piezo 2 and transient receptor potential vanilloid 4 (TRPV4) channels are believed to play important roles in transducing the stimuli evoking the mechanical component of the exercise pressor reflex (Copp et al., 2016; Fukazawa et al., 2023). Likewise, acid-sensing ion channel 1a (ASIC1a) and endoperoxide 4 (EP4) channels are believed to play important roles in transducing the stimuli evoking the metabolic component of the reflex when the exercising muscles are freely perfused (Ducrocq et al., 2020; Yamauchi et al., 2013). In addition, ASIC3 channels are believed to transduce, at least in part, the metabolic component of the reflex when the exercising muscles are ischaemic (Kim et al., 2020; Tsuchimochi et al., 2011).

Controversy exists over the role played by the transient receptor potential vanilloid 1 (TRPV1) cation channel in evoking the metabolic component of the exercise pressor reflex. Determination that a channel on the ending of an afferent fibre can evoke a reflex under investigation often involves three steps. In the case of the TRPV1 channel and the exercise pressor reflex, the first step is to demonstrate that a specific agonist to TRPV1, namely capsaicin, reflexively increases arterial blood pressure and heart rate when injected into the arterial supply of skeletal muscle. This has been successfully and repeatedly demonstrated in several species, including dogs (Crayton et al., 1981; Webb-Peploe et al., 1972), cats (Kindig et al., 2005) and rats (Li et al., 2004; Smith & McQueen, 2001). The second step is to show that a TRPV1 channel agonist stimulates the afferent fibres believed to comprise the sensory arm of the exercise pressor reflex. This too has been demonstrated in dogs (Kaufman et al., 1982), cats (Kaufman et al., 1983) and rats (Hoheisel et al., 2004). The third and most important step is to demonstrate that the exercise pressor reflex is attenuated when the TRPV1 channel is blocked with an antagonist. Attempts to demonstrate the third step have been controversial, with one laboratory reporting that TRPV1 antagonists attenuated the exercise pressor reflex (Mizuno et al., 2011; Smith et al., 2010) and another reporting that TRPV1 antagonists had no effect on the reflex (Ducrocq et al., 2019; Kindig et al., 2005; Tsuchimochi et al., 2010)

Genetic engineering using the CRISPR–Cas9 technique (Kalamakis & Platt, 2023) enables one to generate a functional knockout of the TRPV1 channel and, as a consequence, has provided us with an additional step to the ones described above. We tested the hypothesis that the exercise pressor reflex is attenuated in rats in which the TRPV1 channel has been rendered non-functional by removing 26 base pairs on exon 3 (Stocker & Sullivan, 2023). We demonstrated the functional knockout of the TRPV1 channel by showing that capsaicin, injected into the systemic circulation of the decerebrated rat, did not evoke a reflex increase in arterial pressure. In addition, we demonstrated the functional knockout of the channel by showing that capsaicin did not evoke inward currents in isolated dorsal root ganglion neurons innervating the rats’ gastrocnemius muscle. Our findings show that functional knockout of the TRPV1 channel had no effect on the exercise pressor reflex in decerebrated unanaesthetized rats regardless of whether the arterial supply to their hindlimb muscles was either patent or occluded.

## Methods

### Ethical approval

All procedures were conducted in accordance with the National Institutes for Health guidelines, with the approval of the Pennsylvania State University College of Medicine Institutional Animal Care and Use Committee (IACUC), and according to journal policies and regulations on animal experimentation. Adult TRPV1 channel wild-type (TRPV1^+/+^), TRPV1 channel heterozygous (TRPV1^+/−^) and TRPV1 channel knockout (TRPV1^−/−^) rats were used and were bred from TRPV1^+/−^ parents. The rats were generated by the Gene Editing Resource Center of the Medical College of Wisconsin. TRPV1^−/−^ rats were developed on a Sprague–Dawley background and had a 26 bp deletion in exon 3. As described by Stocker & Sullivan (2023), TRPV1^−/−^ rats were produced by pronuclear injection into one cell Sprague–Dawley embryos of a CRISPR–Cas9 ribonucleoprotein targeting the sequence CTGCGATCATAGAGCCTCGG. TRPV1^−/−^ rats have a normal resting arterial blood pressure, heart rate and glomerular filtration rate but lack both TRPV1 immunofluorescence in the dorsal root ganglion (DRG) and renal afferent nerve responses to intrarenal infusion of the TRPV1 agonist, capsaicin (Stocker & Sullivan, 2023). For the experiments, male and female rats were used randomly. All rats were housed within the central animal facility of the Pennsylvania State University College of Medicine, had access to food and water *ad libitum*, and were exposed to a 50:50 light–dark cycle. All attempts were made to minimize animal discomfort and pain. Data were obtained from 69 rats, of which 52 contributed to the *in vivo* experiments, eight to the *in vitro* patch clamp experiments, and nine to the western blot experiments. Of the 52 rats used in the *in vivo* experiments, not every rat contributed data to the each of the manoeuvres (i.e. contraction, stretch and capsaicin injection) that we employed.

### *In vivo* experimental procedures

#### Surgical procedures.

On the day of the experiment, rats were anaesthetized with a mixture of 3% isoflurane and 100% oxygen as described previously (Ducrocq et al., 2019). The surgical procedures were started only when the corneal reflex stopped and when pinching the hindpaw did not produce a withdrawal reflex. Blood arterial *P*_O2_ (100–150 mmHg), *P*_CO2_ (35–40 mmHg) and pH (7.35–7.45) were regularly monitored and kept within the physiological range. The trachea was cannulated, and the lungs were mechanically ventilated (model 683; Harvard Apparatus, Holliston, MA, USA). The left and right common carotid arteries were cannulated to record blood pressure (P23XL; Gould-Statham Instruments, Los Angeles, CA, USA). Similarly, the right jugular vein was cannulated to inject fluid and drugs into the systemic circulation. In six rats, a snare was placed on the femoral artery and vein approximately 1 cm proximal to the superficial epigastric artery. When tightened, the snare occluded the femoral circulation to the hindlimb muscles. The pelvis was clamped to hold the rat securely in place. In experiments in which the hindlimb muscles were contracted, the left popliteal fossa was opened to expose and isolate the tibial nerve, which was hooked with a bipolar stainless steel electrode. The triceps surae muscles were isolated. The hindpaw was secured to the experimental table, and the head of the rat was secured using a customized stereotaxic unit. The calcaneus bone was severed, and with its attached Achilles tendon, was connected to a force transducer (FT03; Grass Instrument, Quincy, MA, USA). A precollicular decerebration was performed (Dobson & Harris, 2012; Smith et al. 2001) and all neural tissue rostral to the plane of section was removed. The isoflurane anaesthesia was then terminated, and the lungs were subsequently ventilated with room air. Body temperature was maintained around 37°C using a heating lamp. The experimental protocol was initiated after the lungs were ventilated with room air for 45–60 min. At the end of the experiment, the decerebrated rats were killed by intravenous injection of 3 ml of a supersaturated KCl solution and the chest was opened.

#### Contraction of the triceps surae muscles.

Baseline tension of the triceps surae muscles was set at ~100 g and the tibial nerves was electrically stimulated with a Grass Instruments S88 stimulator connected to a PSIU 6 constant current unit. The motor threshold was determined by progressively increasing the current of a single pulse (0.01 ms) applied to the tibial nerve until a muscle twitch was observed. The stimulator output was then set at a current intensity that was 1.5 times motor threshold. The tibial nerve was stimulated for 30 s at 40 Hz (0.01 ms pulse duration) to statically contract the hindlimb muscles. After a recovery period of at least 10 min, the contraction procedure was repeated to verify that the pressor response was reproducible. To control for the possibility that tibial nerve stimulation electrically activated the axons of the group III and IV afferents, which evoke the exercise pressor reflex, the rat was subjected to neuromuscular blockade by intravenous injection of pancuronium bromide (1 mg/ml; 0.2 ml), and the tibial nerve was stimulated for 30 s at 40 Hz (0.01 ms), with the highest current used to evoke contraction. If an increase in blood pressure of more than 5 mmHg was observed after the rat was subjected to neuromuscular blockade, then the data were excluded. In these decerebrated rats, we then examined the effect on blood pressure and heart rate of the TRPV1 agonist 8-methyl-*N*-vanillyl-6-nonenamide (capsaicin, 0.5 *μ*g in 250 *μ*l) injected into the systemic circulation via the right carotid artery. The person performing the experiments was blinded as to whether the rat was a TRPV1^+/+^, TRPV1^+/−^ or TRPV1^−/−^.

#### Tendon stretch.

In five rats in which we determined the effects of static contraction and capsaicin injection on blood pressure and heart rate, we also measured the effect of tendon stretch on these dependent variables. Baseline tension of the triceps surae muscles was set at 100 g, after which the calcaneal tendon was stretched for 30 s by turning a rack and pinion so that the tension developed approximated that evoked by static contraction. In these experiments, the femoral artery was patent; 5 min before tendon stretch, pancuronium bromide (1 mg/ml; 0.2 ml) was injected intravenously to prevent movement. The person performing the experiments was blinded about whether the rat was a TRPV1^+/+^, TRPV1^+/−^ or TRPV1^−/−^.

#### Drug preparation.

Stock solution of capsaicin (Sigma-Aldrich, St Louis, MO, USA) was made by dissolving 10 mg of powder in 0.1 ml ethanol and then adding a drop of Tween 80, which is an emulsifier. Afterwards, 9.9 ml saline was added. The solution was stirred and gently heated to avoid visible flakes, after which saline (40 ml) was added. The final solution was made on the day of the experiment by diluting the stock solution with saline.

#### Data analysis.

Tension and arterial blood pressure signals were amplified (Gould Universal and Pressure Processors; Gould-Statham Instruments), displayed and recorded at 1 kHz using an analog-to-digital converter (Micro1401 MKII; Cambridge Electronic Design, Cambridge, UK) and its associated commercially available software (Spike2; Cambridge Electronic Design). Heart rate was calculated electronically beat by beat from the arterial pressure wave and expressed as beats per minute. To determine the pressor response to 30 s of static contraction, we calculated the difference between the peak mean arterial pressure and its corresponding baseline value. The blood pressure index (BPI) was calculated by integrating the area under the curve during the contraction period and then subtracting from this value the area under the curve measured 30 s before the contraction. Using a similar method, we calculated the change in peak tension produced by contraction, as well as the tension–time index (i.e. the equivalent of the blood pressure index for tension).

To test for differences between TRPV1^+/+^, TRPV1^+/−^ and TRPV1^−/−^ rats with patent femoral arteries, we used a one-way between groups ANOVA. To test for differences between the groups of rats when their femoral arteries and veins were first patent and then were subsequently occluded, we used a one between (i.e. TRPV1^+/+^ *vs*. TRPV1^−/−^), one within (i.e. freely perfused *vs*. occluded) ANOVA. If an overall significant difference was found between the main effects, Tukey’s *post hoc* test was performed to assess significance between any of the two mean values. The data are expressed as the mean ± standard deviation (SD). The criterion for statistical significance was *P* < 0.05.

### *In vitro* experimental procedures and electrophysiology

#### DRG neurons labelling and isolation.

Dorsal root ganglion (DRG) neurons were isolated as described previously (Farrag et al., 2017). Briefly, the neurons innervating the triceps surae muscles were identified using the retrograde fluorescent neuronal tracer DiI (1,1*’*-dioctadecyl- 3,3,3*’*,3*’*-tetramethylindocarbocyanine perchlorate; Thermo Fisher Scientific, Waltham, MA, USA).

Three days prior to DRG neuron isolation, 100 *μ*l of DiI (3% in dimethyl sulfoxide (DMSO)) was injected into the triceps surae muscles. On the day the neurons were isolated, the rats were anaesthetized with CO_2_ and then decapitated. The lumbar DRGs (L_4_–L_5_) were dissected and the connective tissue was then cleared in ice-cold Hanks’ balanced salt solution (Sigma-Aldrich). The ganglia were then enzymatically dissociated in Earle’s balanced salt solution (Sigma-Aldrich) containing collagenase type D (0.6 mg/ml; Roche Diagnostics Corp., Indianapolis, IN, USA), trypsin (0.4 mg/ml, Worthington, Lakewood, NJ, USA), and DNase (0.1 mg/ml; Alfa Aesar, Ward Hill, MA, USA) in a water bath for 50 min at 35°C. Afterward, the neurons were vigorously shaken and centrifuged twice for 6 min at 50 *g* and then placed in minimum essential medium (Thermo Fisher Scientific) supplemented with 10% fetal bovine serum (VWR, Radnor, PA, USA), 1% glutamine and 1% penicillin–streptomycin (both from Thermo Fisher Scientific). Finally, the neurons were plated onto 35 mm poly-l-lysine-coated dishes and placed overnight in a humidified incubator (5% CO_2_–95% air) at 37°C.

#### Whole-cell patch-clamp.

TRPV1 currents were recorded from DiI-labelled DRG neurons employing the whole-cell variant of the patch-clamp technique. The recordings were accomplished with an Axopatch 200B amplifier (Molecular Devices, San Jose, CA, USA), which was analog filtered at a frequency of 2 kHz (3 dB, 4-pole low-pass Bessel filter), and digitized (2–5 kHz) with custom-designed F6 software (developed by Stephen R. Ikeda, NIH/NIAAA) equipped with an 18-bit AD converter board (ITC-18, HEKA Instruments, Inc., Bellmore, NY, USA) employing Igor Pro (Wave-Metrics, Inc., Lake Oswego, OR, USA). The recording pipettes, made of borosilicate glass (King Precision Glass, Claremont, CA, USA), were pulled with a P-97 micropipette puller (Sutter Instrument Co., Novato, CA, USA). Both cell membrane capacitance and pipette series resistance were electronically compensated (80–85%). A custom-designed gravity-fed perfusion system (Lu & Ikeda, 2016) was used, and TRPV1 currents were evoked by switching external recording solutions with 10 *μ*M capsaicin solution for 3 s (TRPV1^+/+^ and TRPV1^+/−^) or 15 s (TRPV1^−/−^). ATP (10 *μ*M) was applied as a positive control. The holding potential was kept at −80 mV and the current density was calculated from the peak TRPV1 current amplitude divided by the membrane capacitance. The person performing the experiments was blinded as to whether the neurons were obtained from TRPV1^+/+^, TRPV1^+/−^ or TRPV1^−/−^ rats.

#### Recording solutions and drug.

The external solution used to record capsaicin-induced currents consisted of (in mM): NaCl 140, KCl 5.4, HEPES 10, MgCl_2_ 1, CaCl_2_ 5, glucose 10 and TTX 0.0003, to pH 7.4 with NaOH. The pipette solution consisted of (in mM): *N*-methyl-d-glucamine (NMG) 80, tetraethyl ammonium hydroxide (TEA-OH) 20, EGTA 11, HEPES 10, CaCl_2_ 1, CsCl 20, CsOH 40, Mg-ATP 4, Na_2_GTP 0.3 and creatine phosphate 14, to pH 7.2 with CH_3_SO_3_H.

Stock solutions (10 mM) of capsaicin (Sigma-Aldrich) and ATP were prepared with ethanol and distilled water, respectively, and diluted in the external solution to their final concentration (10 *μ*M) on the day of the experiment.

#### Data analysis.

For data and statistical analysis, Igor Pro 6.0 and Prism 6.0 (GraphPad Software, San Diego, CA, USA) were used, respectively. The data are expressed as means ± standard deviation, and were evaluated using a one-way between-groups ANOVA. The criterion for statistical significance was *P* < 0.05. Graphs and current traces were generated with Igor Pro 6.0 and iDraw (Indeeo, Palo Alto, CA, USA) software.

#### Protein analysis.

Western blot experiments were performed with the Wes system (Protein Simple, San Jose, CA, USA) using manufacturer-provided microplate, buffers, horseradish peroxidase-conjugated secondary mouse antibody, total protein module, and reagents. The microplate was loaded with protein concentrations of 0.6 and 0.9 *μ*g/*μ*l for EP4 and ASIC1, respectively. Primary antibodies anti-EP4 (cat. no. sc-55 596, Santa Cruz Biotechnology, Dallas, TX, USA) and anti-ASIC1 (cat. no. N271/44, NeuroMab, Davis, CA, USA) were employed at 1:25 and 1:100, respectively. EP4 and ASIC1 signals were normalized to the total protein loading for each individual sample.

## Results

### Functional verification of the knockout

**Figure 1.**
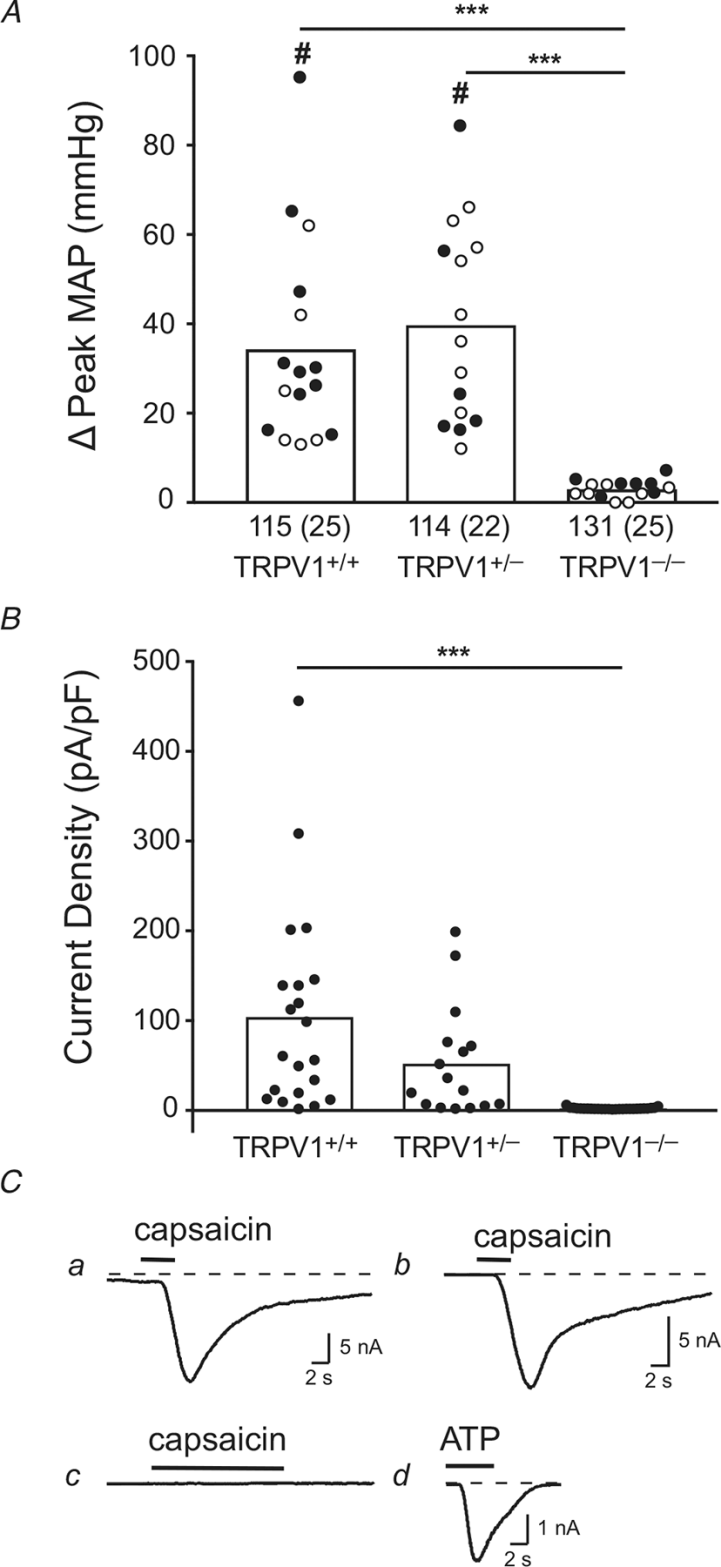
*In vivo* and *in vitro* effects of capsaicin *A*, summary *in vivo* data for the peak pressor responses to intra-carotid injection of capsaicin (0.5 *μ*g in 250 *μ*l) in 16 TRPV1^+/+^ rats, 15 TRPV1^+/−^ rats and 15 TRPV1^−/−^ rats. Filled circles represent data from males and open circles represent data from females. # signifies that Δ peak mean arterial pressure (MAP) value was significantly greater than its corresponding baseline value (*P* < 0.0001), which is shown below each vertical bar. Values in parentheses are standard deviations. Baseline values were not significantly different from each other (*P* > 0.116). Horizontal lines connect means that are significantly different from each other (***P < 0.001). *B*, summary data for *in vitro* currents densities evoked by capsaicin (10 *μ*M) in 21 DRG neurons isolated from TRPV1^+/+^ rats, 16 DRG neurons taken from TRPV1^+/−^ rats and 27 DRG neurons taken from TRPV1^−/−^ rats. *C*, examples of individual currents evoked by capsaicin (10 *μ*M) and/or ATP (10 *μ*M) applied at the horizontal bar. *a*, recording from a neuron isolated from a TRPV1^+/+^ rat. *b*, recording from a neuron isolated from a TRPV1^+*/*−^ rat. *c*, recording from a neuron isolated from a TRPV1^−/−^ rat. *d*, neuron that did not respond to capsaicin. Subsequently, ATP (10 *μ*M) was applied as a positive control.

In *in vivo* experiments, we found that capsaicin (0.5 *μ*g), injected into the aorta via the carotid artery, evoked significant pressor responses in both TRPV1^+/+^ (34.3 ± 23.0 mmHg; *n* = 16) and TRPV1^+/−^ rats (39.6 ± 22.0 mmHg; *n* = 15), but had only a trivial effect on arterial pressure in TRPV1^−/−^ rats (2.9± 1.9 mmHg; n = 15; Fig. 1*A*). Similarly, in *in vitro* experiments, we measured the TRPV1 current density following application of capsaicin (10 *μ*M) to isolated DRG neurons innervating the gastrocnemius muscles. Capsaicin evoked strong inward currents in DRG neurons isolated from the TRPV1^+/+^ (*n* = 21) and TRPV1^+/−^ rats (*n* = 16), but did not evoke currents in neurons isolated from TRPV1^−/−^ rats (*n* = 27; *P* < 0.0001; Figs 1*B* and *C*). The magnitude of the capsaicin-induced current density in the TRPV1^+/+^ neurons was not significantly greater than the magnitude of the current density in TRPV1^+/−^ neurons (*P* = 0.085).

### Responses to static contraction and tendon stretch – patent femoral arteries

**Figure 2.**
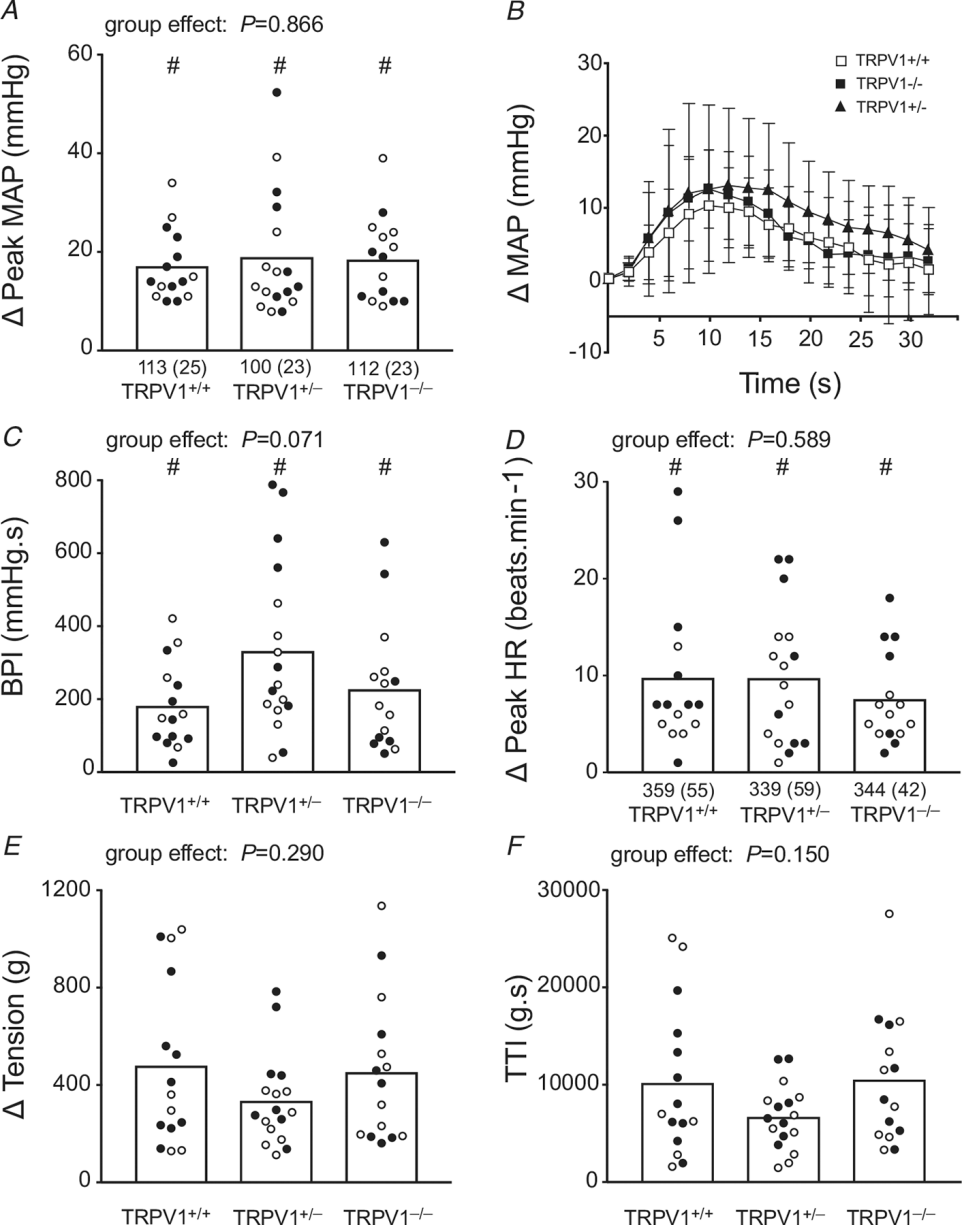
Summary data for the *in vivo* responses to static contraction in TRPV1^+/+^, TRPV1^+/−^ and TRPV1^−/−^ rats *A* and *B*, there was no significant difference between the peak pressor responses to static contraction (*A*) and time courses of the average changes in mean arterial pressure (MAP) during static contraction (*B*) among the three groups of rats. *C–F*, likewise, there were no significant differences among the three groups of rats for blood pressure indices (BPI) (*C*), peak heart rates (HR) (*D*), peak tensions developed by the statically contracting triceps surae muscles (*E*) and tension time indices (TTI) developed by the contracting muscles (*F*). Baseline mean values and standard deviation (parentheses) for MAP and HR are shown below the open columns. # signifies that values are significantly different from their corresponding baseline values (*P* < 0.001). Filled circles represent values obtained from males and open circles represent values obtained from females. There were no significant differences between baseline values for either MAP or HR.

We measured the cardiovascular reflex responses to static contraction in 15 TRPV1^+/+^ rats (9 males and 6 females), 17 TRPV1^+/−^ rats (8 males and 9 females) and 15 TRPV1^−/−^ rats (7 males and 8 females). The femoral artery was patent in each of these 47 rats. Overall, the peak pressor response, the blood pressure index and the cardioaccelerator response were not different among the three groups of rats (Fig. 2*A*, *C* and *D*). In addition, the time courses of the pressor responses to contraction were not different among the three groups (Fig. 2*B*). From a statistical point of view, there was no difference in either the tension time indices (TTIs) or the peak tensions between the three groups of rats (Fig. 2*E* and *F*); these measures of contractility, however, appeared lower in the TRPV1^+/−^ rats than those in the TRPV1^+/+^ and TRPV1^−/−^ rats. Even so, they do not support the hypothesis that removal of TRPV1 channels reduces the exercise pressor reflex because there was no difference in the reflex between the three groups of rats.

**Figure 3.**
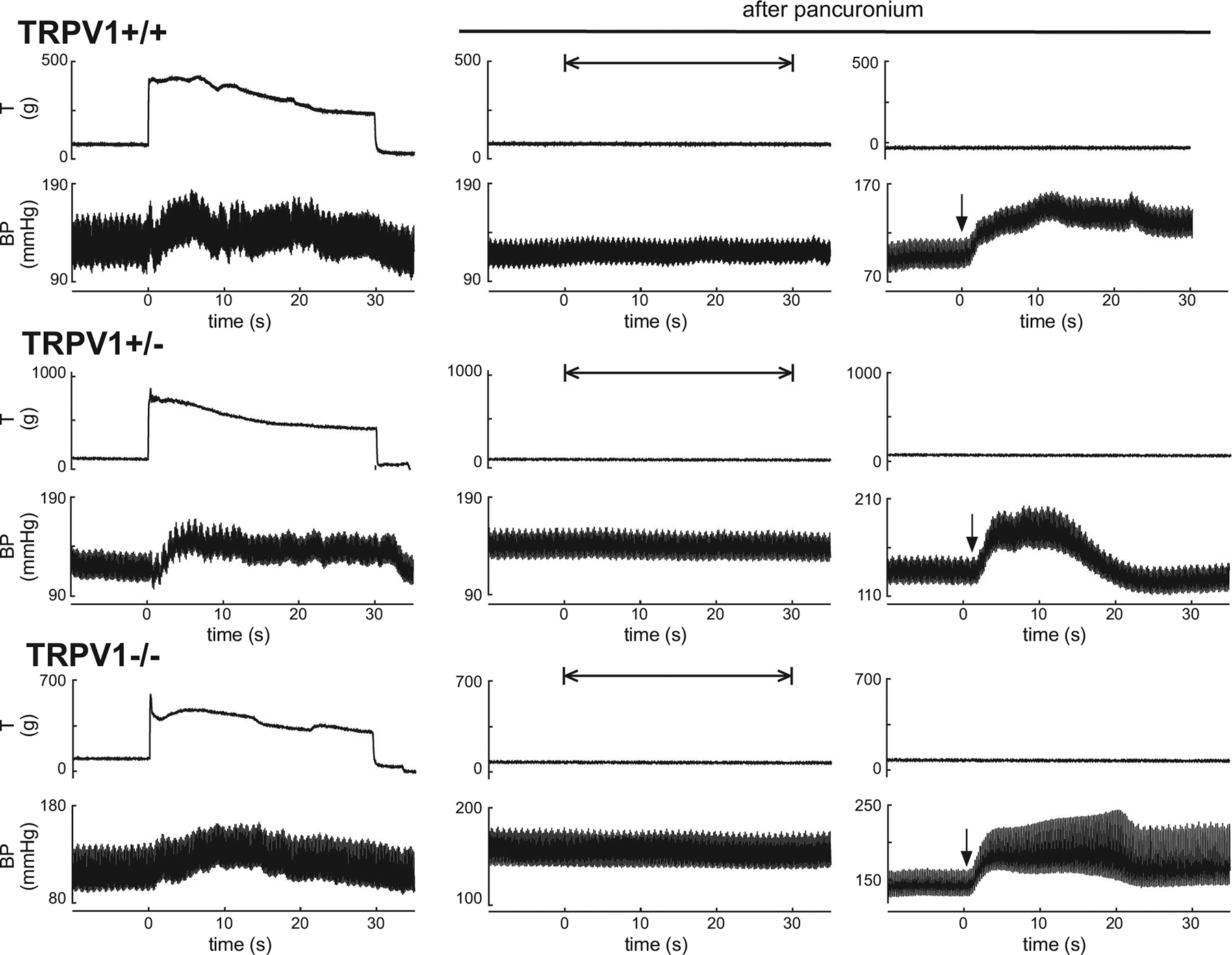
Individual examples of the pressor responses to static contraction in TRPV1^+/+^, TRPV1^+/−^ and TRPV1^−/−^ rats Traces on the left show the pressor responses to contraction evoked by stimulating the tibial nerve at 40 Hz, 0.01 ms and 1.5 times motor threshold. Traces in the middle show that lack of a pressor response to stimulating (40 Hz, 0.01 ms and 1.5 times motor threshold) the tibial nerve when the rats were subjected to neuromuscular blockade with pancuronium. Traces on the right show the pressor responses to stimulating the tibial nerve at 40 Hz, 0.5 ms and 70 times motor threshold. This was done to show that the preparation could still generate a pressor response after it was subjected to neuromuscular blockade. Arrows signify the onset of electrical stimulation. Abbreviations: BP, blood pressure; T, tension.

We next subjected the rats to neuromuscular blockade with pancuronium bromide and electrically stimulated the tibial nerve with the same currents, pulse width and frequency as those used to contract the triceps surae muscle. Stimulation of the tibial nerve in the rats subjected to neuromuscular blockade had no effect on arterial pressure (TRPV1^+/+^: 1.3 ± 1.8 mmHg, *n* = 15; TRPV1^+/−^: 1.9 ± 2.3 mmHg, *n* = 17; TRPV1^−/−^: 1.2 ± 2.0 mmHg, *n* = 15; Fig. 3). This finding indicated that the pressor responses to contraction were not caused by electrical stimulation of group III and IV axons within the tibial nerve.

**Figure 4.**
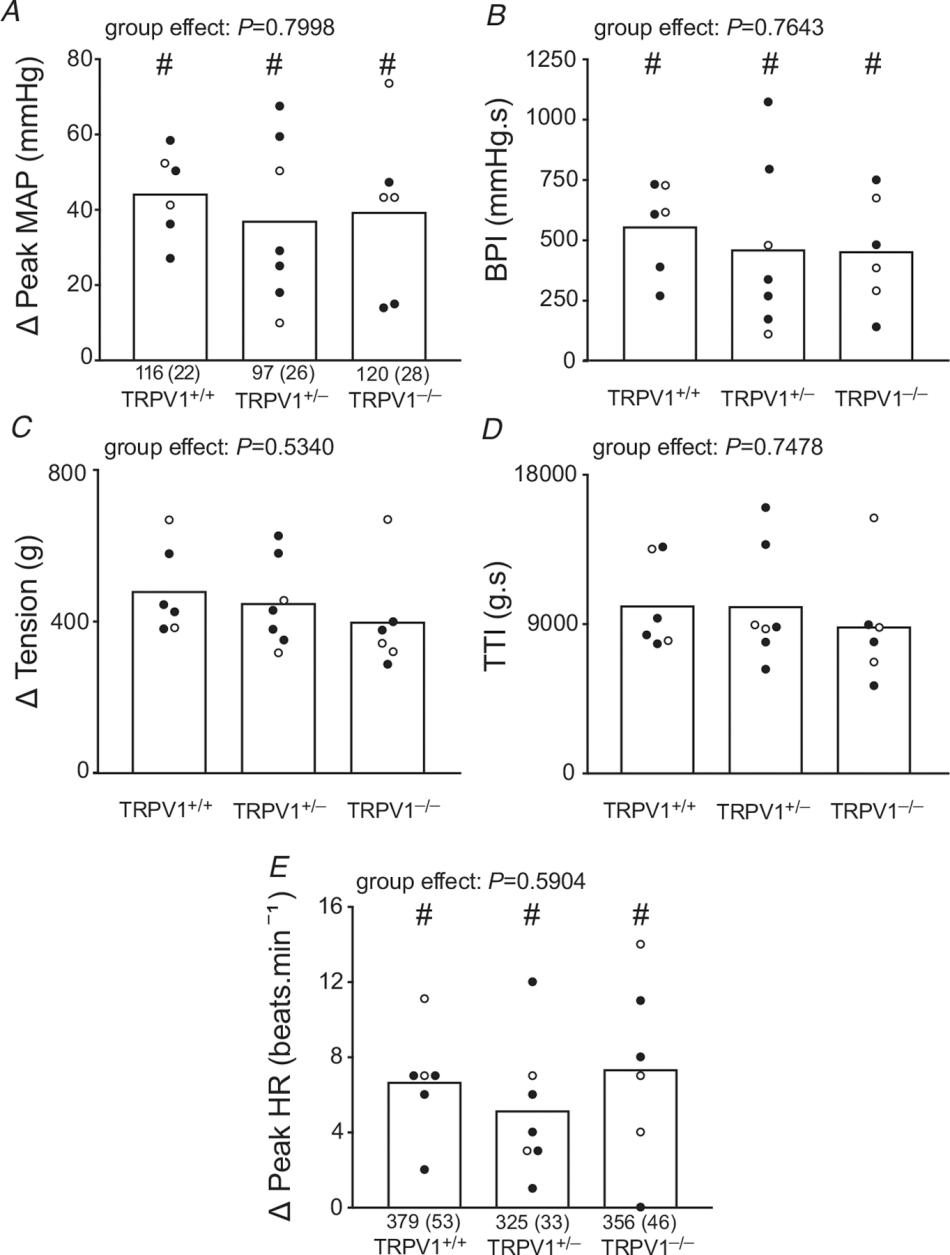
Summary data for the *in vivo* responses to tendon stretch in TRPV1^+/+^, TRPV1^+/−^ and TRPV1^−/−^ rats *A–E*, there were no significant differences among the three groups of rats for peak mean arterial pressure (MAP) (*A*), blood pressure indices (BPI) (*B*), peak tension (*C*), tension time indices (TTI) and peak heart rates (HR) (*D*). Baseline mean values and standard deviation (parentheses) for MAP, HR and tension are shown below the open columns. Filled circles represent values obtained from males and open circles represent values obtained from females. # signifies that values are significantly different from their corresponding baseline values (*P* < 0.005). There were no significant differences between baseline values for either MAP or HR.

We also measured the cardiovascular reflex responses to tendon stretch in six TRPV1^+/+^ rats (4 males and 2 females), in seven TRPV1^+/−^ rats (5 males and 2 females) and six TRPV1^−/−^ rats (3 males and 3 females). The pressor and cardioaccelerator responses to stretch did not differ between the three groups (Fig. 4).

The cardiovascular responses to static contraction and tendon stretch between the TRPV1^+/+^ rats and the TRPV1^+/−^ rats did not differ significantly from each other. These findings led us to conclude that further investigation using the TRPV1^+/−^ rat would offer no useful information about the role played by the TRPV1 channel in evoking the metaboreceptor component of the exercise pressor reflex. Therefore, in the experiments to be described next, we report our findings only for the TRPV1^+/+^ and the TRPV1^−/−^ rat.

### Responses to static contraction-occluded femoral arteries and veins

**Figure 5.**
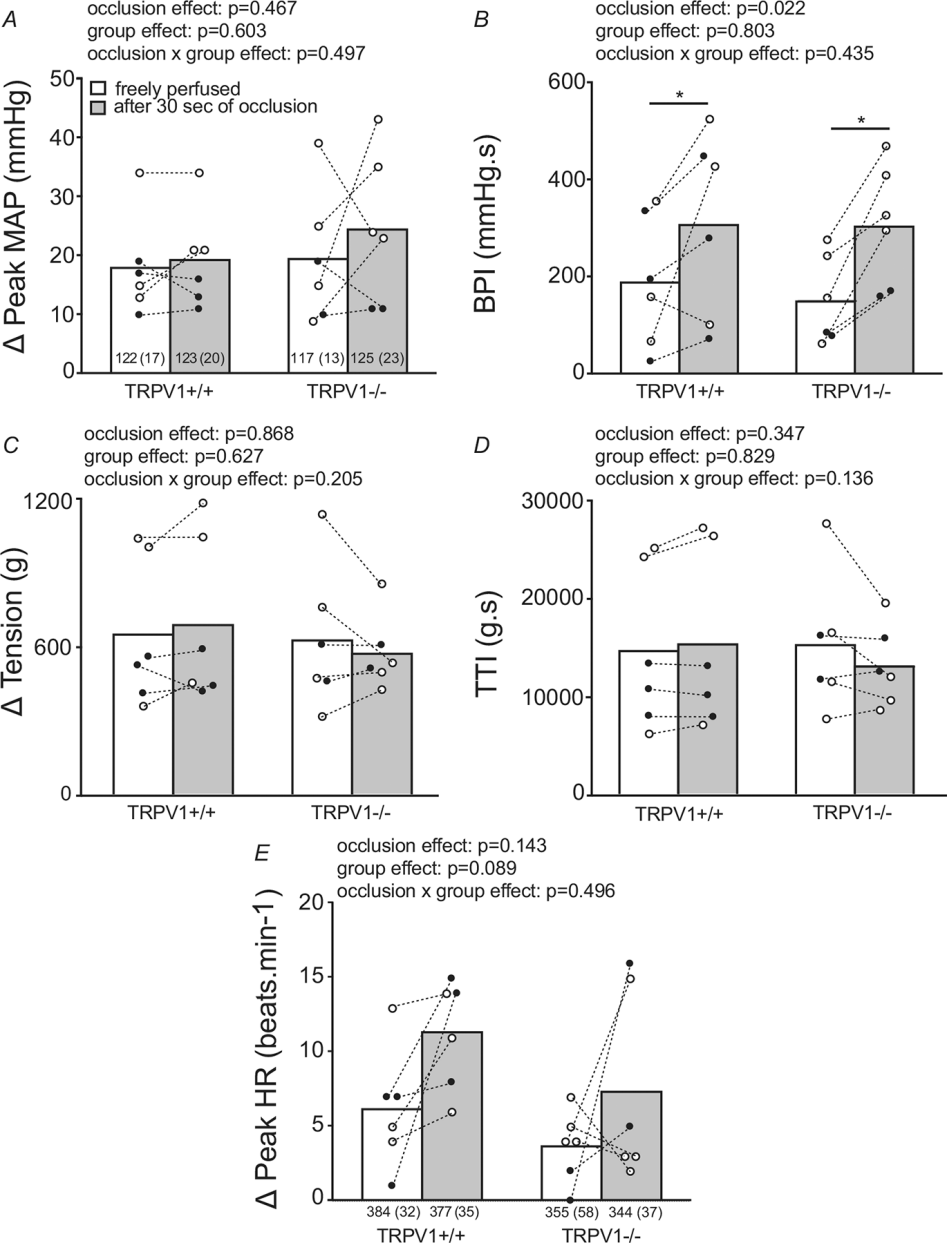
Summary data for *in vivo* responses to static contraction in TRPV1^+/+^ and TRPV1^−/−^ rats with or without occluded femoral artery and vein Baseline values (standard deviation) for MAP, tension and HR are shown inside the columns. Note that the pressor responses to contraction integrated over the 30 s contraction period (i.e. BPI) were equally exaggerated in the TRPV1^+/+^ and the TRPV1^−/−^ rats. Dashed lines connect the responses of individual rats with or without circulatory occlusion. Filled circles represent values obtained from males and open circles represent values obtained from females. There were no significant differences between baseline values for either MAP or HR. Horizontal lines connect mean values that are significantly different from each other (*P < 0.04).

In six of the TRPV1^+/+^ rats (3 males and 3 females) and in six of the TRPV1^−/−^ rats (2 males and 4 females), we compared their cardiovascular responses to static contraction when their femoral arteries and veins were patent with their responses to contraction when their femoral arteries and veins were occluded. Overall, we found no differences between the two groups of rats in their peak pressor (*P* = 0.497), their integrated pressor (i.e. BPI; *P* = 0.435), their peak tension (*P* = 0.205), TTI (*P* = 0.136) (Fig. 5*A*–*D*) or their cardio-accelerator responses to contraction (*P* = 0.143) (Fig. 5*E*). Nevertheless, we found that within each of the two groups of rats that the contraction-induced BPI while the femoral artery and vein were occluded was significantly greater than the contraction-induced BPI while the femoral artery and vein were patent (*P* = 0.022; Fig. 5*B*). The contraction-induced peak pressor response while the femoral artery was occluded was not significantly greater than the contraction-induced peak pressor response while the femoral artery was patent (*P* = 0. 497; Fig. 5). The contraction data obtained from these 12 rats while their femoral arteries and veins were patent are included in Fig. 2.

### EP4 and ASIC1 expression in DRG neurons

**Figure 6.**
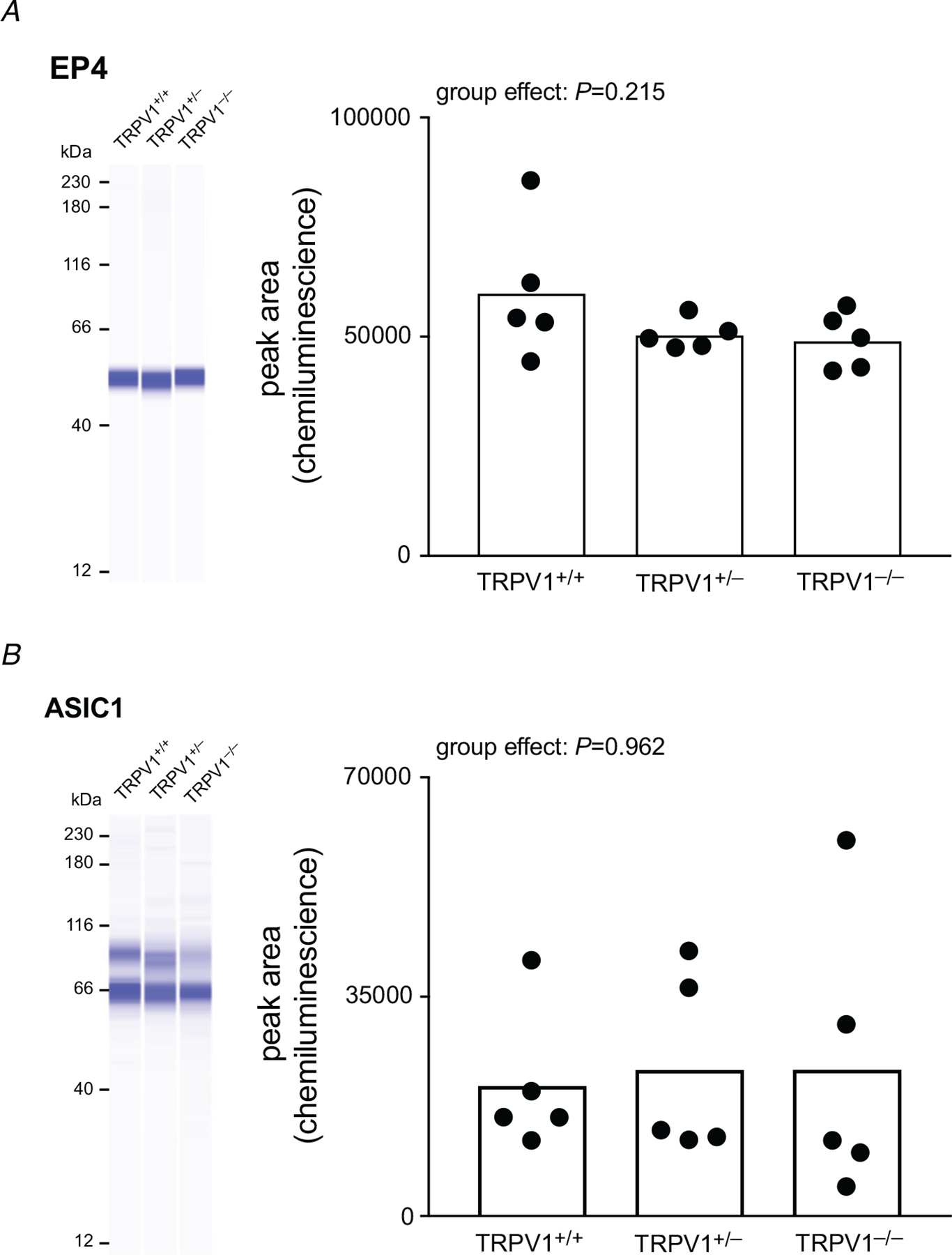
Acid-sensing ion channel 1 (ASIC1) and endoperoxide receptor 4 (EP4) antibody expression in TRPV1^+/+^, TRPV1^+/−^ and TRPV1^−/−^ rats *A* and *B*, representative digitally generated blot and quantification of EP4 (*A*) and ASIC1 (*B*) in dorsal root ganglion (DRG) tissue isolated from the three groups of rats. For EP4 blots, the bands are shown at 53 kDa. For ASIC1 the bands shown at 64 and 89 kDa represent the non-glycosylated and glycosylated forms of ASIC1, respectively. There is no significant change in the expression of EP4 (*P* = 0.323) and ASIC1 (*P* = 0.387) between the three groups.

In a separate group of rats (5 for each group), expression levels of EP4 and ASIC1 in L4–L5 DRG neurons were measured to assess the possibility of a compensatory mechanism following the absence of TRPV1. Using western blots, we found that EP4 and ASIC1 were not upregulated in the TRPV1^−/−^ rats (Fig. 6). Each of these proteins has been shown to play a role in evoking the afferent arm of the exercise pressor reflex (Ducrocq et al., 2020; Yamauchi et al., 2013).

## Discussion

We have shown that CRISPR-induced functional knockout of the TRPV1 channel in decerebrated rats whose femoral arteries were either patent or occluded had no effect on the magnitude of the exercise pressor reflex. This finding is consistent with previous ones from our laboratory showing that pharmacological blockade of TRPV1 had no effect on this reflex in either cats (Kindig et al., 2005) or rats (Ducrocq et al., 2019; Tsuchimochi et al., 2011). *In vivo* evidence of TRPV1 channel knockout was provided by our showing that capsaicin injected into the systemic circulation evoked a pressor response in both TRPV1^+/+^ and TRPV1^+/−^ rats, but did not evoke a pressor response in TRPV1^−/−^ rats. *In vitro* evidence of a functional knockout was provided by our showing that capsaicin application to DRG neurons innervating the gastrocnemius muscles evoked inward currents in neurons isolated from both TRPV1^+/+^ and TRPV1^+/−^ rats, but did not evoke inward currents in neurons innervating these muscles in TRPV1^−/−^ rats. Our findings in rats contrast with a recent report in mice that knockout of TRPV1 attenuated exercise pressor reflex (Li & Garry, 2020).

One explanation for this contrast may in part be related to differences in the distribution of ASICs and TRPV1 channels between rats and mice. Specifically, Leffler et al. (2006) found that transient, ASIC-like inward currents recorded from isolated lumbar dorsal root ganglion neurons were less frequently observed in the mouse than in the rat; moreover, the dorsal root ganglion neurons that did show transient currents in mice had lower conductances compared to those in rats. These findings suggest that mice have fewer ASICs on thin fibre afferents than do rats. Based on these findings, we speculate that in mice TRPV1 channels play a more significant role in transducing increases in hydrogen ion concentrations than do these channels in rats. We note with interest that ASIC1a channels have been shown to play an important role in evoking the exercise pressor reflex in rats when the femoral arteries are patent (Ducrocq et al., 2020), whereas ASIC3 channels play an important role in in evoking the reflex in preparations when the femoral arteries are occluded (Kim et al., 2020; Tsuchimochi et al., 2011). In mice, we speculate that these roles may be relegated to TRPV1 channels.

In our experiments, the pressor responses to capsaicin injected into the carotid artery of TRPV1^+/−^ rats were similar to the pressor responses to capsaicin in their TRPV1^+/+^ counterparts. This is surprising because the expression of TRPV1 channels in the TRPV1^+/+^ rats would be expected to be greater than that of TRPV1 channels in the TRPV1^+/−^ rats We can only speculate as to why the pressor responses to capsaicin in the TRPV1^+/+^ rats were not greater than the pressor responses to capsaicin in the TRPV1^+/−^ rats. One possibility involves the limited ability of group IV afferents to discharge at high frequencies. The consequence of this limitation is that the capsaicin-induced stimulation of TRPV1 channels supplying group IV afferents in the TRPV1^+/−^ rats maximized their ability to generate action potentials. The addition of more TRPV1 channels may not have been able to increase the discharge of the group IV afferents supplying TRPV1^+/+^ rats. This speculation is consistent with our *in vitro* finding that current density evoked by capsaicin in DRG neurons taken from TRPV1^+/+^ rats was not significantly different from the current density evoked by capsaicin in DRG neurons taken from TRPV1^+/−^ rats. A second possibility is that spinal and medullary processing of group IV afferent input equated the pressor responses to capsaicin in the two groups of rats.

In our experiments in rats, the peak pressor response to static contraction while the femoral artery and vein were occluded was not significantly greater than the peak pressor response to contraction while the femoral artery and vein were patent. This finding replicates that reported previously by our laboratory in rats (Tsuchimochi et al., 2010), and it might appear at first glance to contrast with the commonly held belief reported in cats that circulatory occlusion amplifies the magnitude of the metaboreceptor component of the exercise pressor reflex (Coote et al., 1971; McCloskey & Mitchell, 1972; Stebbins & Longhurst, 1989). The contrast can be explained by our finding in rats that the integrated pressor response (i.e. the BPI) to contraction while the femoral artery and vein were occluded was significantly and substantially greater than the integrated pressor response to contraction while the femoral artery and vein were patent. Moreover, the magnitude of the increase in the BPI induced by circulatory occlusion was the same in the TRPV1^+/+^ rats as it was in the TRPV1^−/−^ rats. The importance of this finding is that it provides no support for TRPV1 channels playing a role in evoking the exercise pressor reflex even when its metaboreceptor component was exacerbated by an occluded blood supply to the contracting muscles.

The nature of the stimulus to TRPV1 channels during exercise is unknown, but two important candidates are decreases in pH and increases in temperature. In electrophysiological experiments on human embryonic kidney cells transfected with the TRPV1 channel, inward currents were not evoked until the pH was below 6.0 (Cortright et al., 2001; Nilius et al., 2007). Likewise, inward currents were not evoked from these transfected cells until temperature was increased to over 42°C (Cortright et al., 2001; Nilius et al., 2007). From a physiological point of view, these threshold levels for pH and temperature do not occur in muscle during exercise even under severely fatiguing conditions (Febbraio et al., 1994; Street et al., 2001). Evidence has been presented, however, that the threshold temperature of the TRPV1 channel can be lowered to physiological levels by its exposure to bradykinin (Sugiura et al., 2002).

Considerable controversy exists over the role played by TRPV1 channels on the endings of group III and IV muscle afferents in evoking the exercise pressor reflex. One laboratory has found that pharmacological blockade of TRPV1 attenuated the reflex (Mizuno et al., 2011; Smith et al., 2010), whereas our laboratory has found that blockade had no or little effect on the reflex (Ducrocq et al., 2019; Kindig et al., 2005; Tsuchimochi et al., 2010). We believe that the attenuation of the reflex by pharmacological antagonists to TRPV1 channels can be explained by the use of inadequate vehicle controls and off-target effects (Ducrocq et al., 2019). For example, antagonists to TRPV1 are hydrophobic and cannot be placed into solution with saline or water, yet saline was used as the vehicle control for capsazepine and IRTX by Smith et al. (2010) and Mizuno et al. (2011). Moreover, capsazepine, a TRPV1 antagonist, blocks voltage gated Ca^2+^ channels (Docherty et al., 1997), which play an important role in neurotransmitter release. Likewise, ruthenium red, another TRPV1 antagonist used by Smith et al and Mizuno et al., blocks ryanodine channels (Xu et al., 1999), an effect that reduces the muscles’ ability to produce force (Ducrocq et al., 2019), which in turn reduces the stimulus to group III and IV afferents. In addition, ruthenium red blocks TRPA1 channels (Clapham et al., 2005), which have been shown to play a role in evoking the exercise pressor reflex (Koba et al., 2011).

TRPV1 excitotoxins, such as high dose capsaicin and resiniferatoxin, have been used to assess the role played by thin fibre afferents (i.e. mostly group IV) in evoking the exercise pressor reflex in both humans and animals. When excitotoxins are applied to the skin overlying skeletal muscle of humans (Vianna et al., 2018) or cats (Nelson et al., 2004) the exercise pressor reflex is attenuated. Likewise, when injected intrathecally in dogs, the metaboreceptor component of the reflex is attenuated (Mannozzi et al., 2021). The excitotoxin-induced attenuation of the exercise pressor reflex is caused by both the desensitization of thin fibre muscle afferents possessing TRPV1 channels and the depletion of the neurotransmitters released by these afferents (Bevan & Szolcsányi, 1990). In these reports, the attenuation of the reflex cannot be attributed solely to the inactivation of TRPV1 channels. Specifically, individual group IV muscle afferents possessing TRPV1 also possess ASICs (Jankowski et al., 2013; Molliver et al., 2005). In fact, 70% of the lumbar dorsal root ganglion cells that tested positive for ASIC1a also tested positive for TRPV1 (Ugawa et al., 2005). This finding is particularly relevant because selective pharmacological blockade of ASIC1a channels attenuated the exercise pressor reflex in rats (Ducrocq et al., 2020). As a consequence, attenuation of the exercise pressor reflex by a TRPV1 excitotoxin in the experiments described above may have been due to the inactivation of thin fibre muscle afferents possessing ASIC1a channels and not to TRPV1 channels.

Other investigators have demonstrated that TRPV1 channels are not needed to evoke physiological effects. For example, Ugawa et al. (2002) found in humans that capsazepine, a TRPV1 antagonist, did not block the subjects’ report of pain in response to acidic saline (pH 6.0) injected into their skeletal muscles, whereas amiloride, an antagonist to ASICs, did prevent the subjects’ reports of pain to this stimulus. In addition, Seo et al. (2010) found in rats that blockade of TRPV1 with AMG 9810 had no effect on mechanical allodynia induced by a thrombus-induced occlusion of the femoral artery, whereas blockade of ASICs with amiloride and blockade of purinergic 2X receptors with pyridoxalphosphate-6-azophenyl-2′ ,4′ -disulfonic acid attenuated the allodynia. Likewise, Belvisi et al. (2017) found in humans that blockade of TRPV1 had no effect on the frequency of spontaneous coughing, whereas blockade of P2X3 channels reduced it (Abdulqawi et al., 2015). Last, Tucker & Stocker (2016) found that knockout of TRPV1 channels in rats had no effect on both drinking and vasopressin secretion induced by intravenous infusion of 2M NaCl (i.e. hypernatraemia).

Interpretation of our findings is subject to three limitations. The first, and most important, involves the issue of compensation in the knockout model. Specifically, we cannot exclude the possibility that the TRPV1^−/−^ rat develops other receptor proteins on group III and IV muscle afferents that, in turn, compensate for the loss of functional TRPV1 channels. In part, we assessed the likelihood of this possibility by seeing if functional knockout of TRPV1 caused an upregulation of two other proteins in the dorsal root ganglion neurons that play an important role in the elicitation of the exercise pressor reflex. Specifically, we found that functional knockout of TRPV1 did not result in an upregulation of either the endoperoxide 4 protein (Yamauchi et al., 2013) or the ASIC1 protein (Ducrocq et al., 2020). The second limitation involves our method of contracting the triceps surae muscles. Electrically stimulating the tibial nerve in our experiments activated group I and II muscle afferents (i.e. Golgi tendon organs and spindles) as well as *α*-motoneurons. Group I and II afferents are not activated electrically when the triceps surae muscles are contracted by stimulating the ventral roots (Smith et al., 2001). Stimulation of group I and II afferents, however, has no reflex effect on cardiovascular function (Hodgson & Matthews, 1968; Waldrop et al., 1984), so it is not conceivable that they played a role in evoking the exercise pressor reflex in our experiments. The third limitation is that the knockout of TRPV1 channels in our experiments was global, and consequently may have altered central neural transmission of information controlling cardio-vascular function during muscle contraction. Evidence, for example, has been presented that TRPV1 channels are found on the terminal endings of unmyelinated baroreceptors synapsing in the nucleus tractus solitarius of the medulla (Andresen et al., 2012), a finding which raises the possibility that baroreflex modulation of the exercise pressor reflex may have been altered in our experiments on TRPV1^−/−^ rats. Controversy exists over whether TRPV1 or ASIC3 plays a major role in evoking the metabolic component of the exercise pressor reflex that is exaggerated by circulatory occlusion (Ducrocq et al., 2019; Kindig et al., 2005; Li & Garry, 2020; Mizuno et al., 2011; Tsuchimochi et al., 2011). Our findings in TRPV1^−/−^ rats strongly suggest that this channel is not needed to evoke the metabolic component of the exercise pressor reflex. In contrast, there is substantial evidence that ASIC3 plays an important role in evoking this metabolic component. For example, pharmacological blockade of ASIC3 with APETx2 attenuated the muscle metaboreflex induced by circulatory occlusion in decerebrated rats (Tsuchimochi et al., 2011). Likewise, CRISPR-induced knockout of ASIC3 in decerebrated rats also attenuated the muscle metaboreflex (Kim et al., 2020). We believe that investigations into the nature of the muscle metaboreceptor should focus on ASIC3 instead of TRPV1. Nevertheless, ASIC3 may not function as the sole receptor evoking the metaboreflex. For example, there is evidence that the P2X3 channel and the endoperoxide 4 channel may also contribute to this reflex (Xing et al., 2013; Yamauchi et al., 2013).

## Supplementary Material

Supplementary File

## Data Availability

The data that support the findings of this study are available from the corresponding author upon reasonable request.

## Key points

- A reflex arising in contracting skeletal muscle contributes to the increases in arterial blood pressure, cardiac output and breathing evoked by exercise.
- The sensory arm of the reflex comprises both mechanoreceptors and metaboreceptors, of which the latter signals that blood flow to exercising muscle is not meeting its metabolic demand.
- The nature of the channel on the metaboreceptor sensing a mismatch between supply and demand is controversial; some believe that it is the transient receptor potential vanilloid 1 (TRPV1) channel.
- Using genetically engineered rats in which the TRPV1 channel is rendered non-functional, we have shown that it is not needed to evoke the metaboreflex.
