# Supplementary material for: Functional knockout of the TRPV1 channel has no effect on the exercise pressor reflex in rats
Source: J Physiol. Author manuscript; Available in PMC 2025 Apr 30. (PMC12042824; doi:10.1113/JP285267)
Supplement: Supplementary File [file NIHMS2061498-supplement-Supplementary_File.pdf]

The Journal of Physiology

Functional Knockout of the TRPV1 Channel Has No Effect on the Exercise Pressor Reflex in Rats.

Laura Anselmi, Guillaume P. Ducrocq, Victor Ruiz-Velasco, Sean D Stocker, Shannon P Higgins, and Marc P Kaufman
DOI: 10.1113/JP285267

Corresponding author(s): Laura Anselmi (lanselmi@pennstatehealth.psu.edu)

The following individual(s) involved in review of this submission have agreed to reveal their identity: Mary G Garry (Referee #3)

Review Timeline:

Submission Date:	02-Mar-2023
Editorial Decision:	20-Apr-2023
Resubmission Received:	10-Jul-2023
Editorial Decision:	11-Sep-2023
Revision Received:	25-Sep-2023
Accepted:	11-Oct-2023

Senior Editor: Harold Schultz

Reviewing Editor: Igor Fernandes

Transaction Report:

(Note: With the exception of the correction of typographical or spelling errors that could be a source of ambiguity, letters and reports are not edited. Depending on transfer agreements, referee reports obtained elsewhere may or may not be included in this compilation. Referee reports are anonymous unless the Referee chooses to sign their reports.)

Dear Dr Anselmi,

Re: JP-RP-2023-284636 "Functional Knockout of the TRPV1 Channel Has No Effect on the Exercise Pressor Reflex in Rats." by Laura Anselmi, Guillaume P Ducrocq, Victor Ruiz-Velasco, Sean D Stocker, Shannon P Higgins, and Marc P Kaufman

Thank you for submitting your manuscript to The Journal of Physiology. It has been carefully assessed by a Reviewing Editor and 3 Referees and relevant feedback from their reports is copied below for your information. We regret to inform you that your manuscript is not considered acceptable for publication in The Journal of Physiology.

In view of reviewer comments, we feel that - even if the specific concerns described below were addressed - the overall priority of this manuscript would not be sufficient for publication in its current form. Although we are unable to offer any further consideration of your manuscript at this stage, we hope that the points raised in the reports will be helpful. You are, of course, welcome to resubmit your manuscript again at a later date if you feel that the concerns raised can be adequately addressed.

We kindly request that you inform your co-authors of this decision as soon as possible.

We know that our decision will be disappointing. We recognize the effort and dedication put into your work, and we deeply appreciate your interest in The Journal of Physiology. Currently, we only accept about 25% of submitted manuscripts, and as a consequence many studies containing good quality research must be declined.

We are sorry that we are unable to communicate a more favourable outcome, but hope that our decision on this occasion will not prevent you from submitting future work to The Journal of Physiology.

Yours sincerely,

Harold D Schultz
Senior Editor
The Journal of Physiology
https://jp.msubmit.net
http://jp.physoc.org
The Physiological Society
Hodgkin Huxley House
30 Farringdon Lane
London, EC1R 3AW
UK
http://www.physoc.org
http://journals.physoc.org

EDITOR COMMENTS

Reviewing Editor:

Thank you for submitting your work to the Journal of Physiology. Unfortunately, the reviewers raised several methodological and interpretative concerns that prevented the manuscript from being further considered for publication in the journal.

Senior Editor:

The manuscript has been evaluated by three external reviewers and summarized by a reviewing editor. Unfortunately, there were a number of serious concerns raised that preclude the ability to continue to assess the paper without significant changes in methodology, verification of the animal model, and interpretation of the data, among other important concerns. We hope the authors can use these comments in the progression of this work and future studies.

REFEREE COMMENTS

Referee #1:

Anselmi and colleagues aimed to test the hypothesis that the exercise pressor reflex is attenuated in rats in which the TRPV1 channel has been rendered nonfunctional by removing 26 base pairs on exon 3. The authors found that functional knockout of the TRPV1 channel had no effect on the exercise pressor reflex in decerebrated unanesthetized rats regardless of whether the arterial supply to their hindlimb muscles was either patent or occluded. Of note, the authors do not offer any

explanation for these contrasting findings. I have the following comments for the authors to consider.

- A major concern is related to the rationale of the present study. The authors need to provide a stronger justification on how the present approach could show a completely different output. Furthermore, the controversy is not between two labs only. There are a plethora of studies involving different species (i.e., murine, rats, cats, dogs, and humans) suggesting a role for TRPV1 in contributing to EPR via its metabolic afferent arm. The authors need to reconcile this information in the introduction section to provide a stronger justification for the present study. Please, provide the caveats in the literature.

- Another major concern: Why didn't the authors used a more stablished model for TRPV1 -/- animal? More than 300 studies in the literature used animals from Jackson Laboratory, developed by Professor Julius. The validation study of the present animal model still under peer review process and it would be important to provide more information, including skeletal muscle mass, function and fiber type (for example, fiber type can affect the exercise pressor reflex).

- The authors need to provide blood pressure and heart rate responses to increasing doses of capsaicin in the present animal model.

- Please, Tables 1 and 2 need to be displayed in a Figure format showing individual responses. It seems that tendon stretch did NOT evoke a significant change in heart rate when compared to its baseline value. Please, clarify this point first before comparing delta responses between groups.

***

Referee #2:

This manuscript describes novel in vivo and in vitro studies, using genetically engineered rats, that were undertaken to investigate the role of the Transient Receptor Potential Vallanoid 1 (TRPV1) channel in the exercise pressor reflex (EPR). Specifically, several series of experiments were undertaken using TRPV1 knock out (KO), heterozygous and wild type rats. Having established that TRPV1-KO rats do not display an increase in blood pressure in response to systemic administration of capsaicin, the manuscript presents data which indicate that the EPR evoked by electrical stimulation of the tibial nerve to contract the triceps surae of decerebrate, anesthetized animals is not mediated by the TRP1V receptor. This supports previous work originating from the same research group. Furthermore, functional knockout of the TRPV1 channels did not alter blood pressure responses to muscle contraction during circulatory occlusion, indicating that TRPV1 channels are not responsible for the EPR in response to heightened metaboreceptor input. I enjoyed reading this paper and the authors deserve congratulations for their rigorous methodological approach.

General Comments

The Introduction provides a brief overview of background in the research area. Experimental details are described well, and data appears to have been carefully collected. The Discussion contains appropriate speculation on the significance / importance of the findings and includes a sound explanation for hitherto equivocal findings relating to the role TRPV1 channels in the EPR. Moreover, there is some consideration of limitations of the study and future directions.

Overall, this manuscript is well written and presents novel insight and valid conclusions that are relevant to understanding the afferent pathway of the EPR. There is, however, some scope for refinement and improvement.

Major comments

1. Pg 4. For the benefit of reader with no background in the areas of KO models, a basic overview of CRISPR-Cas technology would improve the Introduction.

2. Pg 6. Surgical Procedures. What is meant by 'selected experiments' in relation to placing a snare around the femoral artery and vein? What criteria was used for 'selection'? Also, for the purposes of transparency, please indicate how many animals had a snare placed around the artery and vein. Were data from all these animals included in the analysis/results?

3. Pg 7. What was the rationale for administering capsaicin systemically, via the carotid artery, rather than using a more localized approach to the hind limb vasculature. In other words, have the authors considered the possibility that functional KO of TRPV1 receptors in the heart and elsewhere may contribute to the attenuated pressor response to systemic capsaicin.

4. Pg 7. Please provide details about monitoring and maintenance of anaesthesia. Also, please provide details about steps taken to monitor and maintain arterial blood gasses within a physiological range

5. Pg 7. contraction of the triceps surae muscles. Data from how any experiments were excluded, based on the stated criteria (i.e., an increase in BP or more than 5 mmHg after the rat was paralyzed)?

6. The structure of the Discussion is occasionally difficult to follow. For example, the purpose of paragraphs on Pg 14 and 15 which addressing the potential stimuli to TRPV1 channels during exercise is not clear. In other words, this manuscript, and others from the same group (Kindig et al., 2005; Tsuchimochi et al., 2010; Ducrocq et al., 2019), indicate that TRPV1

channels do not contribute to the EPR. I suggest some refocusing on the insight that the current Manuscript provides into mechanisms of the EPR.

Minor comments

1. Pg 10. N = 15 mmHg appears to be a typing error.

2. Pg 5. Animals. Please indicate the total number of rats used in the experiments

3. Pg 8. Drug preparation. For clarity, explain the purpose of adding a drop of Tween 80.

4. Pg 11. Please indicate if responses to tendon stretch were studied in subsets of the animals used to study effects of capsaicin and static contraction OR if this protocol was performed using separate animals.

5. Pg 12. Similarly, please indicate if responses to static contraction -occluded femoral were investigated in same or different groups.

***

Referee #3:

The studies by Anselmi et al.were intended to determine whether the Trpv1 channel has a role in the exercise pressor reflex in rats. This is similar to a paper from the same group in 2019 (Ducrocq et al, 2019; DOI: 10.1152/ajpregu.00174.2019) in which it is concluded that the Trpv1 channel does not have a role in the exercise pressor reflex in rats. There are multiple and serious concerns regarding this manuscript which will be outlined below.

In the present study, the authors have added the use of a Trpv1 global knock out rat. Unfortunately, there is no genotypic characterization of this rat model. Instead, a paper (in submission) is cited with data not shown. There is some phenotypic data presented but all of it could be explained by modifications not directly due to the deletion of Trpv1 particularly in a global knock out model. A well characterized, conditional and targeted knock out would offer greater insight. The authors did not explore what other genes may be dysregulated in the absence of Trpv1 and, therefore, the conclusions cannot be strictly limited to an effect of Trpv1 deletion. Study of an uncharacterized model lacks appropriate scientific rigor especially for a well regarded journal.

There is also concern with the high variability in the data. Because of this variability, statistical significance is unlikely to be achieved (regardless of treatment) and the results and conclusions may be confounded. For example, peak MAP responses to capsaicin administration vary in WT rats from ~15mmHg to 95 mmHg. The average response in the WT animals was 34.3 {plus minus}23.0 mmHg (Fig. 1A) and this was in a large animal population (n=16). This draws into question the quality of the preparation and placement of the pressure transducer. Moreover, the data in which tibial nerve stimulation and stretch is performed is also highly variable (Figs 2 and 4). The authors may have identified the source of some variability as they indicate that animals that had a MAP response of greater than 5mmHg (to tibial nerve stimulation) in paralyzed rats were eliminated from the study. The fact that MAP is elevated in paralyzed rats supports the notion that tibial nerve stimulation directly activates afferent neurons and, therefore, this method does not reliably measure the exercise pressor reflex. Further, the need to eliminate animals points to the variability in the stimulus parameters and the quality of the preparation as a whole. It will be important for the authors to indicate the number of rats eliminated for this reason and the details involved in each animal. It is also important to note that the preparation of the tibial nerve (which is used for both stimulation and conductance of the signals from the contracting skeletal muscle) has the potential to alter the conductance of the primary afferent neurons in this nerve. Such a possibility may be supported by the fact that the observed response to stretch is significantly greater than the response to tibial nerve stimulation. This is also very likely a source of significant variability in this preparation. Stimulation of the ventral root (which carries only motor neurons) activates muscle contraction. In turn, this muscle contraction stimulates the afferent neurons within the contracting muscle. When the ventral roots are stimulated, the afferent neurons are fully intact and are not impacted by the nerve preparation procedures. This method of stimulation is well established and mimics the exercise pressor reflex and should be used to reduce variability in these studies.

The authors peform 30 sec femoral artery occlusion after which they measure blood pressure and heart rate responses to tibial nerve stimulation. What is the purpose of this occlusion? Is it intended to mimic peripheral artery disease? If so, these authors have several manuscripts in which they indicate that 72 hours of occlusion is needed to mimic this condition. Additionally, the authors have previously published that "three minutes of acute femoral artery occlusion did not augment the pressor response compared with freely perfused limb during static contraction")Tsuchimochi et al, 2010; doi: 10.1152/ajpheart.00141.2010) which is in contrast to the data they present here. In the current manuscript, these data (during occlusion) add no useful information and should be removed. As stated above, the rationale for this occlusion is unclear. Moreover, this data also has remarkable variability. A significant response is only seen when BPI is calculated. What is the rationale for the use of this integration? Lastly, the response to stretch is not reported. Why is this?

The fact that pancuronium bromide was needed before passive stretch is performed suggests that the decerebration is incomplete. The authors need to address this point.

The discussion is confusing and offers information that is not relevant to the role of the Trpv1 channel to the exercise

pressor reflex. For example, the authors offer significant discussion regarding the well established role of the Trpv1 in nociception. Do the authors suggest that activation of the exercise pressor reflex is noxious? Do they further suggest that the activation of the exercise pressor reflex elicits hyperalgesia or allodynia?

There is also a section in the discussion regarding the homozygous and heterozygous rats, the latter of which display unpredicted findings. This discussion is either poorly written or illogical and needs to be addressed.

The statistical analyses seem inappropriate. Why not use a two-way ANOVA with repeated measures when the design calls for it?

The authors conclude (in Key Points Summary) that "these data suggest that Acid Sensing Ion Channels play an important role in evoking the metaboreflex, which in turn elicits exaggerated cardiovascular response to exercise in peripheral artery disease, heart failure, and hypertension". In fact, there were no data to support any of these statements within this manuscript and such a conclusion should be removed.

The authors state as a goal that they will address the controversy surrounding Trpv1 and the exercise pressor reflex but, instead, they seem to avoid the issue. For example, there is evidence that deletion of Trpv1 sensitive afferent neurons in dog (Mannozzi et al., 2021; DOI: 10.1152/ajpregu.00129.2021) and rat (Smith et al., 2005; DOI: 10.1161/01.CIR.0000162473.10951.0A.) significantly decreases the blood pressure response to exercise and metabolic stimulation. These papers are not acknowledged or discussed. The authors reference a paper by Li and Garry (2020; doi: 10.1113/JP277602) in which a significant decrease in the pressor reflex in Trpv1 null mice is observed, conflicting with the findings of the current manuscript. The authors do little to address the differences in these studies but this discussion is important to the stated goals of this manuscript. It is also important to note that the current authors published two papers (Tsuchimochi et al, 2010; doi: 10.1152/ajpheart.00141.2010 and Tsuchimochi et al., 2011 doi: 10.1113/jphysiol.2011.217851) demonstrating that the response to capsaicin is significantly increased following 72 hrs of femoral artery ligation. It would be important for these findings to be integrated into the current findings.

Minor Issues

The use of the precollicular decerebration model should cite Smith et al., 2001.

Are both the femoral artery and vein occluded in this preparation?

There is no methodology provided for the administration of capsaicin in Figure 1. Were the animals decerebrate in these experiments?

Overall, the goals and objectives of this manuscript are unclear and the findings seem incremental. The experimental methods employed seem to have remarkably high variability which limits the interpretation of the data. The discussion does little to incorporate these findings with those previously published (even from the same authors).

Confidential Review 02-Mar-2023

RESPONSES TO THE REFEREES

Referee #1:

Comment: A major concern is related to the rationale of the present study. The authors need to provide a stronger justification on how the present approach could show a completely different output.

Response: The purpose of our study was to shed light on the controversy about the role played by the TRPV1 channel in evoking the exercise pressor reflex (EPR). Previously, using a pharmacological approach, we showed (i.e., Ducrocq et al.) that TRPV1 did not play a role in evoking the EPR. Specifically, Ducrocq et al found that TRPV1 antagonists (capsazepine, ruthenium red or iodoresiniferatoxin), injected into the arterial supply of contracting muscle did not alter the magnitude of the EPR. In this manuscript, we used an alternative approach, namely genetic engineering, to determine if TRPV1 plays a role in evoking the EPR. This latter approach used CRISPR-Cas 9 technology to generate a functional knockout of the TRPV1 channel in rats. The goal was to use two different approaches to converge on the same conclusion, namely that TRPV1 channels play no role in evoking the EPR. We believe that we have achieved our goal.

Comment: The controversy is not between two labs only. There are a plethora of studies involving different species (i.e., murine, rats, cats, dogs, and humans) suggesting a role for TRPV1 in contributing to the exercise pressor reflex via its metabolic afferent arm. The authors need to reconcile this information in the introduction section to provide a stronger justification for the present study. Please, provide the caveats in the literature.

Response: We agree with the reviewer's comment and as a result we have added a paragraph to the Discussion in which we cite manuscripts that have contributed to the controversy. Previously, in an editorial that we wrote for the American Journal of Physiology, (Stone & Kaufman 2021 AJP: Reg 321: R523-524) we addressed the controversy mentioned by the reviewer. In this editorial we described the studies whose findings have been misinterpreted or over interpreted by both readers and authors.

Comment: Another major concern: Why didn't the authors used a more stablished model for TRPV1 -/- animal? More than 300 studies in the literature used animals from Jackson Laboratory, developed by Professor Julius. The validation study of the present animal model still under peer review process and it would be important to provide more information, including <u>skeletal muscle mass</u>, <u>function and fiber type (for example, fiber type can affect the exercise pressor reflex).</u>

Response: The Jackson Laboratory only sells mice; it does not sell rats. Almost all of the studies investigating the exercise pressor reflex in animals have been performed on rats. The manuscript validating the rat TRPV1 knockout model that we used has now been accepted for publication by the journal *Hypertension*; it has been attached to our revised manuscript. In our experiments, we provided additional data validating this knockout model. Specifically, we demonstrated *in vivo* that capsaicin did not evoke a pressor response in TRPV1-/- rats. Likewise, we demonstrated *in vitro* that capsaicin did not evoke an inward current in dorsal root ganglion cells taken from TRPV1-/- rats. With respect to the reviewer's comment concerning fiber type and muscle mass, these issues are peripheral to our investigation; they do not directly pertain to the issue that we are addressing. Moreover, given that the rats used in our experiments originated from the same genetic background and because only the TRPV1 gene was manipulated by CRISPR, major differences in fiber types are unlikely. We used the standard method of assessing the exercise pressor reflex and found that the absence of functional TRPV1

channels had no effect on its magnitude.

Comment: The authors need to provide blood pressure and heart rate responses to increasing doses of capsaicin in the present animal model.

Response: In the present study capsaicin was used simply as a tool to validate the knocking down of TRPV1 receptor. The concentration of capsaicin that was used for this study (0.5 ug in 200 ul) has been selected from the dose-response experiment published by Tucker and Stocker (2016). They found that 0.5 ug of capsaicin induced the greatest effect on BP. In our model, we found that injection of the same dose (0.5 ug) of capsaicin evoked a large increase of BP in TRPV1+/+ and TRPV1+/- rats, but had no effect on arterial pressure in TRPV1-/- rats. The dose of capsaicin that we used was more than enough to test for the possibility that functional TRPV1 channels were present in the rats used in our experiments.

Comment: Please, Tables 1 and 2 need to be displayed in a Figure format showing individual responses. It seems that tendon stretch did NOT evoke a significant change in heart rate when compared to its baseline value. Please, clarify this point first before comparing delta responses between groups.

Response: Yes, it is true that tendon stretch had minimal effect on heart rate. As requested, we placed the heart rate responses to tendon stretch in a figure format showing individual responses.

Referee #2:

Comment: This manuscript describes novel in vivo and in vitro studies, using genetically engineered rats, that were undertaken to investigate the role of the Transient Receptor Potential Vanilloid 1 (TRPV1) channel in the exercise pressor reflex (EPR). Specifically, several series of experiments were undertaken using TRPV1 knock out (KO), heterozygous and wild type rats. Having established that TRPV1-KO rats do not display an increase in blood pressure in response to systemic administration of capsaicin, the manuscript presents data which indicate that the EPR evoked by electrical stimulation of the tibial nerve to contract the triceps surae of decerebrate, anesthetized animals is not mediated by the TRP1V receptor. This supports previous work originating from the same research group. Furthermore, functional knockout of the TRPV1 channels did not alter blood pressure responses to muscle contraction during circulatory occlusion, indicating that TRPV1 channels are not responsible for the EPR in response to heightened metaboreceptor input. I enjoyed reading this paper and the authors deserve congratulations for their rigorous methodological approach.

The Introduction provides a brief overview of background in the research area. Experimental details are described well, and data appears to have been carefully collected. The Discussion contains appropriate speculation on the significance / importance of the findings and includes a sound explanation for hitherto equivocal findings relating to the role TRPV1 channels in the EPR. Moreover, there is some consideration of limitations of the study and future directions.

Overall, this manuscript is well written and presents novel insight and valid conclusions that are relevant to understanding the afferent pathway of the EPR. There is, however, some scope for refinement and improvement.

Response: We appreciate the reviewers overall evaluation of manuscript. Thank you.

Comment: Pg 4. For the benefit of reader with no background in the areas of KO models, a basic overview of CRISPR-Cas technology would improve the Introduction.

Response: We appreciate the reviewer's concern and have added the following reference: Kalamakis G, Platt RJ. CRISPR for neuroscientists. Neuron. 2023 May 7:S0896-6273(23)00306-9. doi: 10.1016/j.neuron.2023.04.021. Epub ahead of print. PMID: 37201524.

Comment: Pg 6. Surgical Procedures. What is meant by 'selected experiments' in relation to placing a snare around the femoral artery and vein? What criteria was used for 'selection'? Also, for the purposes of transparency, please indicate how many animals had a snare placed around the artery and vein. Were data from all these animals included in the analysis/results?

Response: We agree that the sentence 'selected experiments' does not clearly describe what was done. We simply used a new group of rats in which we applied the femoral arterial and venous snare. We changed the sentence in the manuscript, and indicated how many animals were used for this experiment.

Comment: Pg 7. What was the rationale for administering capsaicin systemically, via the carotid artery, rather than using a more localized approach to the hind limb vasculature. In other words, have the authors considered the possibility that functional KO of TRPV1 receptors in the heart and elsewhere may contribute to the attenuated pressor response to systemic capsaicin.

Response: We administered capsaicin systemically for precisely the reason mentioned by the reviewer. The functional knockout of the TRPV1 channel in our rats was global. If this was in fact the case our validation study would be best served if we injected capsaicin retrogradely into the carotid artery. In that way the injectate would have access to all vascular beds.

Comment: Pg 7. Please provide details about monitoring and maintenance of anesthesia. Also, please provide details about steps taken to monitor and maintain arterial blood gasses within a physiological range.

Response: As requested, we have provided the requested details about anesthesia.

Comment: Pg 7. contraction of the triceps surae muscles. Data from how any experiments were excluded, based on the stated criteria (i.e., an increase in BP or more than 5 mmHg after the rat was paralyzed)?

Response: We have provided the requested data.

Comment: The structure of the Discussion is occasionally difficult to follow. For example, the purpose of paragraphs on Pg 14 and 15 which addressing the potential stimuli to TRPV1 channels during exercise is not clear. In other words, this manuscript, and others from the same group (Kindig et al. 2005; Tsuchimochi et al. 2010; Ducrocq et al. 2019), indicate that TRPV1 channels do not contribute to the EPR. I suggest some refocusing on the insight that the current Manuscript provides into mechanisms of the EPR.

Response: We agree that the structure of the Discussion was difficult to follow. Consequently, we revised it with goal of providing a clearer narrative.

Minor comments

Comment: Pg 10. N = 15 mmHg appears to be a typing error.
Response: Thank you for pointing this out. We have corrected it.

Comment: Pg 5. Animals. Please indicate the total number of rats used in the experiments.
Response: We have now done so.

Comment; Pg 8 Drug preparation. For clarity, explain the purpose of adding a drop of Tween 80.
Response: Tween 80 is a detergent that helps dissolve capsaicin. This has now been stated in the manuscript.

Comment: Pg 11; Please indicate if responses to tendon stretch were studied in subsets of the animals used to study effects of capsaicin and static contraction OR if this protocol was performed using separate animals.
Response: This has now been clarified.

Comment: Pg 12. Similarly, please indicate if responses to static contraction-occluded femoral were investigated in same or different groups.
Response: This has now been clarified.

Responses to Reviewer 3:

Comment: In the present study, the authors have added the use of a Trpv1 global knock out rat. Unfortunately, there is no genotypic characterization of this rat model. Instead, a paper (in submission) is cited with data not shown.

Response: The manuscript which was previously in submission (i.e., Stocker and Sullivan 2023) has now been accepted for publication in the AHA journal *Hypertension.* A PDF of this manuscript is attached.

Comment: There is some phenotypic data presented but all of it could be explained by modifications not directly due to the deletion of Trpv1 particularly in a global knock out model.

Response: *In vivo*, we demonstrated that capsaicin, injected into the aorta, did not evoke a pressor response in the TRPV1-/- rats but did evoke a pressor response in their TRPV1+/+ counterparts. *In vitro*, we demonstrated that capsaicin did not evoke an inward current in TRPV1-/- dorsal root ganglion cells innervating the gastrocnemius muscles, but did evoke an inward current in their TRPV+/+ counterparts. The simplest and best explanation for our findings is that the TRPV1 protein was not functional in the TRPV1-/- rats but was functional in the TRPV1+/+ rats. The reviewer dismissed the phenotypic data that we presented without any mentioning of an alternative explanation. An unbiased and reasonable reviewer would state an alternative explanation that in turn could be assessed for its likelihood.

Comment: A well characterized, conditional and targeted knock out would offer greater insight.

Response: The reviewer is correct, but such a model in rats is not available. More importantly, the lack of a “well characterized, conditional and targeted knockout” in mice did not prevent The Journal of

Physiology from recently publishing a similar manuscript (i.e., Li and Garry 2020 598: 3155-3171). Why does this standard apply to us and not to the manuscript by Li and Garry?

Comment: The authors did not explore what other genes may be dysregulated in the absence of Trpv1 and, therefore, the conclusions cannot be strictly limited to an effect of Trpv1 deletion. Study of an uncharacterized model lacks appropriate scientific rigor especially for a well-regarded journal.

Response: To answer this question, we determined if the concentrations of three other receptor proteins in the L4-L5 dorsal root ganglia were dysregulated by the functional knockout of TRPV1. Using Western Blots, we found that ASIC 1, EP4 and P2X3 were not upregulated in the TRPV1-/- rats. The significance of these "three receptor proteins" is that each has been shown to play a role in evoking the afferent arm of the exercise pressor reflex. Obviously, the number of receptors in the dorsal root ganglia that could be assessed is endless, but we believe that the three measured in our experiments provided an adequate and reasonable assessment of the possibility that another receptor was upregulated to compensate for the TRPV1 functional knockout.

Comment: There is also concern with the high variability in the data. Because of this variability, statistical significance is unlikely to be achieved (regardless of treatment) and the results and conclusions may be confounded. For example, peak MAP responses to capsaicin administration vary in WT rats from ~15mmHg to 95 mmHg. The average response in the WT animals was 34.3 {plus minus}23.0 mmHg (Fig. 1A) and this was in a large animal population (n=16). This draws into question the quality of the preparation and placement of the pressure transducer. Moreover, the data in which tibial nerve stimulation and stretch is performed is also highly variable (Figs 2 and 4).

Response: This is an unreasonable comment. The reflex response to exercise or tendon stretch or almost any other reflex is variable in both animal preparations as well as in humans. In fact, this variability can be seen in two manuscripts recently published in The Journal of Physiology. Specifically, see figure 5 in Li & Garry (598:3155-3171) as well as figure 3 in Butenas et al (600: 2105-2125). The variability shown in these two figures is almost identical to that shown in our figure 2. The reviewer's comment questioning the quality of our preparation and the placement of the pressure transducer is not justified. The transducer was always placed at the level of the heart, and this is now stated in the Methods section of the manuscript.

Comment: The authors may have identified the source of some variability as they indicate that animals that had a MAP response of greater than 5mmHg (to tibial nerve stimulation) in paralyzed rats were eliminated from the study. The fact that MAP is elevated in paralyzed rats supports the notion that tibial nerve stimulation directly activates afferent neurons and, therefore, this method does not reliably measure the exercise pressor reflex.

Response: Baseline arterial blood pressure is not "rock-solid steady" in any mammal under any experimental condition. Follow baseline arterial blood pressure for a few minutes in any animal preparation and you will see that it varies by at least 5 mmHg. More importantly examine the middle panel of Figure 3 in our manuscript. You will see that electrical stimulation of the tibial nerve in paralyzed rats had no effect on blood pressure. This is the appropriate control for the stimulating the tibial nerve in unparalysed rats to show that muscle contraction evoked a reflex increase in arterial pressure. The Journal of Physiology recently published a manuscript by Butenas et al (600: 2105-2125). In this manuscript, the preparation and method of evoking contraction, namely tibial nerve stimulation, was identical to that used in our manuscript. Butenas et al made no mention of the number of paralyzed

rats eliminated because electrical stimulation of the tibial nerve increased arterial pressure by 5 mmHg. Moreover, examination of figures 1 and 4 in the Butenas et al manuscript shows baseline blood pressure varying by more than 5 mmHg.

Comment: The need to eliminate animals points to the variability in the stimulus parameters and the quality of the preparation as a whole. It will be important for the authors to indicate the number of rats eliminated for this reason and the details involved in each animal.

Response: We have given the number of rats eliminated when electrical stimulation of the tibial nerve in paralyzed rats increased arterial blood pressure by more than 5 mmHg.

Comment: It is also important to note that the preparation of the tibial nerve (which is used for both stimulation and conductance of the signals from the contracting skeletal muscle) has the potential to alter the conductance of the primary afferent neurons in this nerve. Such a possibility may be supported by the fact that the observed response to stretch is significantly greater than the response to tibial nerve stimulation. This is also very likely a source of significant variability in this preparation.

Response: The reviewer raised an issue that is impossible to disprove. In other words, how does one prove that preparation of the tibial nerve does not alter conductance of the signals? The reviewer's statement that the preparation of the tibial nerve alters the conductance of the primary afferents should be supported by a reference. Otherwise the statement is nothing more than speculation. Our laboratory has a great deal of experience in preparing the tibial nerve for stimulation. When the preparation of the nerve is compromised, we find that the reflex responses evoked from the hindlimb muscles are attenuated; they are not potentiated. If one rearranges the stimulating electrode or further dissects the tissue nearby the nerve, the reflex responses can be attenuated if the manoeuvre is not done carefully. More importantly, the issue is not the magnitude of the pressor responses to either tendon stretch or contraction. The issue is that the pressor responses to either stimulus were not different across the three groups of rats (i.e., TRPV1+/+, TRPV1+/- and TRPV1-/-) used in our experiments.

Comment: Stimulation of the ventral root (which carries only motor neurons) activates muscle contraction. In turn, this muscle contraction stimulates the afferent neurons within the contracting muscle. When the ventral roots are stimulated, the afferent neurons are fully intact and are not impacted by the nerve preparation procedures. This method of stimulation is well established and mimics the exercise pressor reflex and should be used to reduce variability in these studies.

Response: Multiple times, we have stimulated the ventral roots of decerebrated rats to evoke an exercise pressor reflex (Tsuchimochi et al 2011 J Physiol 589:6173-6189; Yamauchi et al 2014 AJP: Heart 306: H450-454; McCord et al 2011 JAP 111: 971-979) and found that its mean and standard errors were no different than what we have reported when we stimulated the tibial nerve in decerebrated rats. For example, the standard error of the mean for the peak MAP response to contraction for the 15 TRPV1+/+ rats was 1.9. Likewise, the corresponding standard deviation was 7.4. The measures of variance are similar to those that we reported previously when we stimulated the ventral roots. We strongly doubt that stimulating the ventral roots would reduce variability as suggested by the reviewer.

Allow me to circle back to the reviewer's previous comment that preparation of the tibial nerve in our experiments alters afferent conduction. The Reviewer suggested we should have stimulated the ventral roots instead of the tibial nerve. To stimulate the ventral roots, one has to remove the bone covering the spinal cord (i.e., perform a laminectomy), open the spinal meninges thereby losing the cerebrospinal

fluid, separate the ventral roots from the dorsal roots and then sever the ventral roots so their peripheral ends can be stimulated. These surgical procedures are far more invasive and damaging than is isolating the tibial nerve. As a result, primary afferent conductance and its spinal processing is a far more likely to be altered when stimulating the ventral roots than when stimulating the tibial nerve.

Comment: The authors perform 30 sec femoral artery occlusions after which they measure blood pressure and heart rate responses to tibial nerve stimulation. What is the purpose of this occlusion? Is it intended to mimic peripheral artery disease? If so, these authors have several manuscripts in which they indicate that 72 hours of occlusion is needed to mimic this condition.

Response: The purpose of the 30 second femoral artery occlusion is to assess the component of the exercise pressor reflex which is evoked by a mismatch between blood supply and demand in contracting muscles. This is the classic technique first used by Alam & Smirk in 1937 and subsequently used by both Coote et al (1971) and McCloskey & Mitchell (1972). This technique is still used today by investigators performing experiments in both humans and in animals.

The purpose of the 30 second femoral artery occlusion was not to mimic peripheral artery disease, which, in the past, we simulated by occluding the femoral artery for 72 hours before the start of the experiment. Occluding the femoral artery for 72 hours alters the concentration of receptors dorsal root ganglion cells innervating the hindlimb muscles. For example, ASIC3 and P2X3 are upregulated and ASIC1 is downregulated. Occluding the femoral artery for 30 seconds before initiating contraction most likely has no effect on receptor concentrations. The reviewer's concern about peripheral artery disease is unfounded and is irrelevant to our current manuscript. Frankly other than to be obstructive, we see no reason for the reviewer to have even mentioned the issue of peripheral artery disease. The purpose of the 30 second occlusion was to increase the metabolic component of the exercise pressor reflex. The possibility existed that during freely perfused contraction that the metabolic component of the reflex was not strong enough to stimulate TRPV1.

Comment: Additionally, the authors have previously published that "three minutes of acute femoral artery occlusion did not augment the pressor response compared with freely perfused limb during static contraction") Tsuchimochi et al, 2010) which is in contrast to the data they present here. In the current manuscript, these data (during occlusion) add no useful information and should be removed. As stated above, the rationale for this occlusion is unclear. Moreover, this data also has remarkable variability. A significant response is only seen when BPI is calculated. What is the rationale for the use of this integration? Lastly, the response to stretch is not reported. Why is this?

Response: Our previously published statement that "three minutes of acute femoral artery occlusion did not augment the pressor response compared with the freely perfused limb" assessed the peak pressor response to contraction (Tsuchimochi et al 2010). This was clearly stated in the discussion seen by reviewer 3 (please see the last paragraph on page 13 which extends onto page 14), but this individual either chose to ignore it or missed it. The pressor response integrated over the entire 30 second contraction period (the BPI) is a more inclusive and informative measure of the exercise pressor reflex than is the peak response. For example, contraction could evoke an increase in arterial pressure that was transient and then reverted back to or below baseline levels even though the hindlimb muscles were still contracting. The peak response would not reveal this finding, but the BPI would. In our previous publication (i.e., Tsuchimochi et al 2010) which was mentioned by the reviewer, the BPI was not measured.

Comment: The fact that pancuronium bromide was needed before passive stretch is performed suggests that the decerebration is incomplete. The authors need to address this point.

Response: The decerebration was complete. We could easily verify the completeness of the decerebration because we could visualize that all neural tissue rostral to the plane of section which was less than 1 mm rostral to the superior colliculus was gone. Examination of the cranial vault revealed a big gaping hole, which was obvious. We paralyzed the rat with pancuronium to prevent any movement while stretching the calcaneal tendon. Such movement involves muscle contraction, which in turn could contribute to the pressor response attributed to stretch.

Comment: The discussion is confusing and offers information that is not relevant to the role of the Trpv1 channel to the exercise pressor reflex. For example, the authors offer significant discussion regarding the well-established role of the Trpv1 in nociception. Do the authors suggest that activation of the exercise pressor reflex is noxious? Do they further suggest that the activation of the exercise pressor reflex elicits hyperalgesia or allodynia?

Response: This is an obstructive comment, and it should have been recognized as such by the editor. We see nothing wrong in discussing the role played by TRPV1 in nociception. TRPV1 is, after all, the focal point of the manuscript. An important point of our discussion, and one which we made clearly, is that contraction in our experiments most likely did not decrease muscle pH and temperature to be in the range of 5.5 and 42 degrees centigrade, respectively; these are the threshold values needed to stimulate TRPV1 channels. The reviewer's comment about allodynia or hyperalgesia is irrelevant, excessive, and frankly inappropriate because it borders on sarcasm. Nevertheless, we agree that part of our discussion contained a paragraph or two that was irrelevant to the role played by TRPV1 in evoking the exercise pressor reflex. As a result, we have removed the paragraph and replaced it with one describing experiments in which TRPV1 have been either permanently or temporarily inactivated by excitotoxins.

Comment: There is also a section in the discussion regarding the homozygous and heterozygous rats, the latter of which display unpredicted findings. This discussion is either poorly written or illogical and needs to be addressed.

Response: We disagree. The section of our discussion regarding homozygous and heterozygous is neither poorly written nor is it illogical. Neither of the other two reviewers objected to this part of our discussion.

Comment: The statistical analyses seem inappropriate. Why not use a two-way ANOVA with repeated measures when the design calls for it?

Response: We analysed the data shown in figure 5 of the original version of our manuscript using a one between-one within analysis of variance (ANOVA). This analysis was not described in the original Methods section of our manuscript. This has now been corrected. We did in fact analyse the data shown in figures 2 and 4 of the original version of our manuscript with a one-way ANOVA. The baseline values are given for each of the relevant variables. The values for Peak MAP and BPI between the three groups of rats are so similar to each other that there is no possibility that a one between-one within ANOVA would reveal any significant effects. Nevertheless, f the editor insists, we will perform a one between-one within ANOVA.

Comment: The authors conclude (in Key Points Summary) that "these data suggest that Acid Sensing Ion

Channels play an important role in evoking the metaboreflex, which in turn elicits exaggerated cardiovascular response to exercise in peripheral artery disease, heart failure, and hypertension". In fact, there were no data to support any of these statements within this manuscript and such a conclusion should be removed.

Response: We agree and have removed this statement.

Comment: The authors state as a goal that they will address the controversy surrounding Trpv1 and the exercise pressor reflex but, instead, they seem to avoid the issue. For example, there is evidence that deletion of Trpv1 sensitive afferent neurons in dog (Mannozzi et al., 2021; DOI: 10.1152/ajpregu.00129.2021) and rat (Smith et al., 2005; DOI: 10.1161/01.CIR.0000162473.10951.0A.) significantly decreases the blood pressure response to exercise and metabolic stimulation. These papers are not acknowledged or discussed.

Response: We agree with this criticism and have added a paragraph to our discussion in which we describe these manuscripts as well as others. Most importantly, we have explained the problems associated with these manuscripts. For example, Mannozzi et al injected resiniferatoxin, a potent TRPV1 excitotoxin, which prevents thin fiber muscle afferents from responding to any stimulus. The thin fiber afferents that have TRPV1 channels also have many other receptors/channels known to be involved with the elicitation of the exercise pressor reflex. Consequently, resiniferatoxin prevents thin fiber muscle afferents from responding to any metabolic stimulus generated by contracting muscle. Thus, one can attribute a resiniferatoxin-induced attenuation of the exercise pressor reflex to thin fiber muscle afferents, but one cannot attribute the attenuation to TRPV1 channels being stimulated during the exercise bout.

Comment: The authors reference a paper by Li and Garry (2020; doi: 10.1113/JP277602) in which a significant decrease in the pressor reflex in Trpv1 null mice is observed, conflicting with the findings of the current manuscript. The authors do little to address the differences in these studies, but this discussion is important to the stated goals of this manuscript. It is also important to note that the current authors published two papers (Tsuchimochi et al, 2010; doi: 10.1152/ajpheart.00141.2010 and Tsuchimochi et al., 2011 doi: 10.1113/jphysiol.2011.217851) demonstrating that the response to capsaicin is significantly increased following 72 hrs of femoral artery ligation. It would be important for these findings to be integrated into the current findings.

Response: First, we did little to address the differences between our study in TRPV1 knockout rats with the study by Li and Garry in TRPV1 knockout mice because other than the species tested, we have no explanation for the difference. The fact that we stimulated the tibial nerve whereas Li and Garry stimulated the ventral roots cannot explain the difference. Second, we do not doubt femoral arterial ligation for 72 hours increases number of TRPV1 channels in dorsal root ganglion cells as well as increases the pressor responses to capsaicin. Femoral arterial ligation for 72 hours also increases the number of ASIC3 and P2x3 receptors in dorsal root ganglion cells as well as the pressor responses to lactic acid and alpha, beta methylene ATP, agonists to the two receptors. The reviewer failed to mention that blockade of TRPV1 channels with iodoresinaferatoxin failed to attenuate the exercise pressor reflex in rats whose femoral arteries were ligated for 72 hours (Tsuchimochi et al 2011). This is the issue, and the reviewer ignored it.

Minor Issues:

Comment: The use of the precollicular decerebration model should cite Smith et al., 2001.

Response: You are correct. We apologize for the omission. This citation has been added to our manuscript.

Comment: Are both the femoral artery and vein occluded in this preparation?

Response: Yes, both were occluded in our preparation. We omitted this in one sentence and have now corrected it. Thank you for pointing this out to us.

Comment: There is no methodology provided for the administration of capsaicin in Figure 1. Were the animals decerebrate in these experiments?

Response: Yes, capsaicin was administered in decerebrated rats. This has been added to our manuscript.

Comment: Overall, the goals and objectives of this manuscript are unclear and the findings seem incremental. The experimental methods employed seem to have remarkably high variability which limits the interpretation of the data. The discussion does little to incorporate these findings with those previously published (even from the same authors).

Response: We disagree. The goal of our experiments was to determine if the TRPV1 knock out rat displays an exercise pressor reflex. This was clearly stated in the Introduction. We did not incorporate previous findings from our laboratory concerning peripheral artery disease because this pathological state was not the point of our experiments.

1st Revision - Editorial Decision 11-Sep-2023

Dear Dr Anselmi,

Re: JP-RP-2023-285267X "Functional Knockout of the TRPV1 Channel Has No Effect on the Exercise Pressor Reflex in Rats." by Laura Anselmi, Guillaume P. Ducrocq, Victor Ruiz-Velasco, Sean D Stocker, Shannon P Higgins, and Marc P Kaufman

Thank you for submitting your manuscript to The Journal of Physiology. It has been assessed by a Reviewing Editor and by 1 expert referees and we are pleased to tell you that it is acceptable for publication following satisfactory revision.

Please advise your co-authors of this decision as soon as possible.

The referee reports are copied at the end of this email.

Please address all the points raised and incorporate all requested revisions or explain in your Response to Referees why a change has not been made. We hope you will find the comments helpful and that you will be able to return your revised manuscript within 4 weeks. If you require longer than this, please contact journal staff: jp@physoc.org.

Your revised manuscript should be submitted online using the link in your Author Tasks: Link Not Available. This link is accessible via your account as Corresponding Author; it is not available to your co-authors. If this presents a problem, please contact journal staff (jp@physoc.org). Image files from the previous version are retained on the system. Please ensure you replace or remove any files that are being revised.

If you do not wish to submit a revised version of your manuscript, you must inform our journal staff (jp@physoc.org) or reply to this email to request withdrawal. Please note that a manuscript must be formally withdrawn from the peer review process at one journal before it may be submitted to another journal.

TRANSPARENT PEER REVIEW POLICY: To improve the transparency of its peer review process, The Journal of Physiology publishes online as supporting information the peer review history of all articles accepted for publication. Readers will have access to decision letters, including Editors' comments and referee reports, for each version of the manuscript, as well as any author responses to peer review comments. Referees can decide whether or not they wish to be named on the peer review history document.

ABSTRACT FIGURES: Authors are expected to use The Journal's premium BioRender account to create/redraw their Abstract Figures. Information on how to access this account is here: https://physoc.onlinelibrary.wiley.com/journal/14697793/biorender-access.

This will enable Authors to create and download high-resolution figures. If authors have used the free BioRender service, they can use the instructions provided in the link above to download a high-resolution version suitable for publication.

The link provided should only be used for the purposes of this submission. Authors will be charged for figures created on this account if they are not related to this manuscript submission.

LANGUAGE EDITING AND SUPPORT FOR PUBLICATION: If you would like help with English language editing, or other article preparation support, Wiley Editing Services offers expert help, including English Language Editing, as well as translation, manuscript formatting, and figure formatting at www.wileyauthors.com/eeo/preparation. You can also find resources for Preparing Your Article for general guidance about writing and preparing your manuscript at www.wileyauthors.com/eeo/prepresources.

REVISION CHECKLIST:

Check that your Methods section conforms to journal policy: https://jp.msubmit.net/cgi-bin/main.plex?form_type=display_requirements#methods.

Check that data presented conforms to the statistics policy: https://jp.msubmit.net/cgi-bin/main.plex?form_type=display_requirements#statistics.

Upload a full Response to Referees file. To create your 'Response to Referees' copy all the reports, including any comments from the Senior and Reviewing Editors, into a Microsoft Word, or similar, file and respond to each point, using font or background colour to distinguish comments and responses and upload as the required file type.

Please upload two versions of your manuscript text: one with all relevant changes highlighted and one clean version with no changes tracked. The manuscript file should include all tables and figure legends, but each figure/graph should be uploaded as separate, high-resolution files. The journal is now integrated with Wiley's Image Checking service. For further details, see: https://www.wiley.com/en-us/network/publishing/research-publishing/trending-stories/upholding-image-integrity-wileys-image-screening-service.

You may also upload:

- 'Potential Cover Art' for consideration as the issue's cover image
- Appropriate Supporting Information (Video, audio or data set: see https://jp.msubmit.net/cgi-bin/main.plex?form_type=display_requirements#supp).

We look forward to receiving your revised submission.

If you have any queries, please reply to this email and we will be pleased to advise.

Yours sincerely,

Harold D Schultz
Senior Editor
The Journal of Physiology
https://jp.msubmit.net
http://jp.physoc.org
The Physiological Society
Hodgkin Huxley House
30 Farringdon Lane
London, EC1R 3AW
UK
http://www.physoc.org
http://journals.physoc.org

REQUIRED ITEMS

-Author photo and profile. First (or joint first) authors are asked to provide a short biography (no more than 100 words for one author or 150 words in total for joint first authors) and a portrait photograph. These should be uploaded and clearly labelled with the revised version of the manuscript. See Information for Authors for further details.

-You must start the Methods section with a paragraph headed Ethical Approval. A detailed explanation of journal policy and regulations on animal experimentation is given in Principles and standards for reporting animal experiments in The Journal of Physiology and Experimental Physiology by David Grundy J Physiol, 593: 2547-2549. doi:10.1113/JP270818.). A checklist outlining these requirements and detailing the information that must be provided in the paper can be found at: https://physoc.onlinelibrary.wiley.com/hub/animal-experiments. Authors should confirm in their Methods section that their experiments were carried out according to the guidelines laid down by their institution's animal welfare committee, and conform to the principles and regulations as described in the Editorial by Grundy (2015). The Methods section must contain details of the anaesthetic regime: anaesthetic used, dose and route of administration and method of killing the experimental animals.

-Your manuscript must include a complete Additional Information section

-You must upload original, uncropped western blot/gel images (including controls) if they are not included in the manuscript. This is to confirm that no inappropriate, unethical or misleading image manipulation has occurred https://physoc.onlinelibrary.wiley.com/hub/journal-policies#imagmanip These should be uploaded as 'Supporting information for review process only'. Please label/highlight the original gels so that we can clearly see which sections/lanes have been used in the manuscript figures.

-Please include an Abstract Figure file, as well as the figure legend text within the main article file. The Abstract Figure is a piece of artwork designed to give readers an immediate understanding of the research and should summarise the main conclusions. If possible, the image should be easily 'readable' from left to right or top to bottom. It should show the physiological relevance of the manuscript so readers can assess the importance and content of its findings. Abstract Figures should not merely recapitulate other figures in the manuscript. Please try to keep the diagram as simple as possible and without superfluous information that may distract from the main conclusion(s). Abstract Figures must be provided by authors no later than the revised manuscript stage and should be uploaded as a separate file during online submission labelled as File Type 'Abstract Figure'. Please ensure that you include the figure legend in the main article file. All Abstract Figures

should be created using BioRender. Authors should use The Journal's premium BioRender account to export high-resolution images. Details on how to use and access the premium account are included as part of this email.

EDITOR COMMENTS

Reviewing Editor:

The authors have responded adequately to most of the comments - conducting additional experiments and including data to better characterize the model - from the original review of this manuscript. However, the authors should address or edit a few remaining relatively minor issues before a final decision. Here they go:

Key points summary - The last paragraph of the key points summary raises a hypothesis that was not the target of the present investigation. I recommend the authors exclude the sentence or edit it to be exclusively representative of the study.

Abstract - The abstract fails to describe the primary responses reported in the manuscripts - MAP and HR to muscle contraction. Instead of saying that no difference was found in the reflex, I suggest that the authors describe that the hemodynamic responses to muscle contraction did not differ among the groups. The inclusion of the numbers also helps to improve the readability of the abstract. I also recommend mentioning the findings of the additional experiments in the abstract. That will provide a better picture of the model to future manuscript readers.

Introduction - The second paragraph should start with describing the potential receptors and channels in the terminal ending of the afferent fiber responsible for the activation of the metabolic component of the reflex (especially those that the authors targeted in the additional experiments). That would also provide a background and justification for measuring their expression in the dorsal root ganglion. I also believe that the section needs a rationale to justify the inclusion of the responses to stretching. In other words, why investigate the contribution of the TRPV1 in evoking the mechanical component of the reflex? Finally, reviewer 2, although generally satisfied with the responses to his first review, highlighted the need for a little more detail about the approach used to knock out the TRPV1 receptors without referring solely to the article cited by the authors.

Methods - For clarity, in the 'animal' subpart of the section, please insert the total number of animals included in the study. Without it, it is hard to follow certain parts of the methods (for instance, when the authors stated that pancuronium abolished the pressor response to stimulation in 92% of the rats tested), even with the available description of the number of animals in the results section.

Statistical analysis and results - The authors did not report the resting baseline data. Again, for the sake of clarity, a better description of the model, and ultimately, provide an appropriate scenario for data interpretation, I recommend the authors include the data and, in addition to delta responses, conduct a multifactorial ANOVA and report main effects for the hemodynamic responses to capsaicin and tibial nerve stimulation.

Senior Editor:

Thank you for submission of a revised manuscript. We have failed to obtain reviews from the original referees 1 and 3. However, the remaining referee and our reviewing editor have provided some constructive comments for an additional revision. Referee 2, although generally satisfied with the responses to his first review, highlighted the need for a little more detail about the approach used to knock out the TRPV1 receptors without referring **solely** to the article cited by the authors. I suggest this is needed given the unique model. The reviewing editor also has provided other minor comments that should be straightforward to correct. When submitting the revision, please ensure that you provide a copy with changes highlighted.

REFEREE COMMENTS

Referee #2:

I enjoyed reading this resubmission. Concerning replies to points highlighted in my previous review, I thank the authors for their considered responses. Generally, I am satisfied. However, from the perspective of a typical reader, I would like a little more specific detail about the approach used to knockout the TRPV1 receptors without having to refer to an article cited by the authors. Additionally, it appears that numbers of animals reported for some of the analyses has changed from the first submission. The authors have not specifically highlighted these changes - or the reason(s) why - in the 'Responses to referees' document. This is a minor issue. Nevertheless, for transparency, I suggest that authors explain in their response why numbers differ between versions.

END OF COMMENTS

1st Confidential Review **10-Jul-2023**

GENERAL REQUIREMENTS

Comment: Author photo and profile.

Response: As requested, we have included a photo of the first author and her profile.

Comment: You must start the Methods section with a paragraph headed Ethical Approval. Authors should confirm in their Methods section that their experiments were carried out according to the guidelines laid down by their institution's animal welfare committee, and conform to the principles and regulations as described in the Editorial by Grundy (2015). The Methods section must contain details of the anesthetic regime: anesthetic used, dose and route of administration and method of killing the experimental animals.

Response: We have complied with all of the required listed above.

Comment: You must upload original, uncropped western blot/gel images (including controls) if they are not included in the manuscript.

Response: As required, we have uploaded original uncropped western blot images.

Comment: Please include an Abstract Figure file, as well as the figure legend text within the main article file.

Response: As requested, we have included an Abstract Figure and an accompanying legend.

EDITOR COMMENTS

Reviewing Editor

Comment: Key points summary - The last paragraph of the key points summary raises a hypothesis that was not the target of the present investigation. I recommend the authors exclude the sentence or edit it to be exclusively representative of the study.

Response: As recommended, the last sentence of the key points summary has been excluded.

Comment: Abstract - The abstract fails to describe the primary responses reported in the manuscripts - MAP and HR to muscle contraction. Instead of saying that no difference was found in the reflex, I suggest that the authors describe that the hemodynamic responses to muscle contraction did not differ among the groups. The inclusion of the numbers also helps to improve the readability of the abstract. I also recommend mentioning the findings of the additional experiments in the abstract. That will provide a better picture of the model to future manuscript readers.

Response: As recommended, we have added a sentence to the abstract describing the pressor responses to contraction in the three groups of rats. Likewise, we have added a sentence mentioning that genetic manipulation of TRPV1 had no effect on expression levels of ASIC1 or EP4 in the L4 and L5 dorsal root ganglia.

Comment: Introduction - The second paragraph should start with describing the potential receptors and channels in the terminal ending of the afferent fiber responsible for the activation of the metabolic component of the reflex (especially those that the authors targeted in the additional experiments). That would also provide a background and justification for measuring their expression in the dorsal root ganglion. I also believe that the section needs a rationale to justify the inclusion of the responses to stretching. In other words, why investigate the contribution of the TRPV1 in evoking the mechanical component of the reflex? Finally, reviewer 2, although generally satisfied with the responses to his first review, highlighted the need for a little more detail about the approach used to knock out the TRPV1 receptors without referring solely to the article cited by the authors.

Response: As requested, we have added a paragraph describing the potential receptors and channels responsible for the metabolic component of the reflex and have focused on the two targeted in our "additional experiments." We prefer, however, not to add a rationale for performing the stretch experiments described in our manuscript. We performed the stretch experiments because we wanted to characterize completely the newly developed rat TRPV1 knock out model, and having obtained the data felt obligated to report it in our manuscript. Our preference is to include the stretch data with minimal comment. Our description of the "stretch data" is brief, and if we had not reported it, the reviewers probably would have asked for it. Including a stretch protocol in publications investigating the exercise pressor reflex is standard and expected by reviewers and readers. More importantly, the stretch data indicates that the CRISPR-induced knockout of TRPV1 had, as expected, no effect on the rats' ability to transduce and respond to mechanical stimuli. We think that our stretch data can stand alone as presented. Nevertheless, if you believe that the stretch data should be omitted from the manuscript, we would be willing to do so.

Comment: Methods - For clarity, in the 'animal' subpart of the section, please insert the total number of animals included in the study.

Response: The total number of rats contributing data to the study was 69, of which 52 contributed to the *in vivo* experiments, eight contributed to the *in vitro* patch clamp experiments and nine contributed to the Western Blot experiments. This information has been added to the Methods section.

Comment: Statistical analysis and results - The authors did not report the resting baseline data. Again, for the sake of clarity, a better description of the model, and ultimately, provide an appropriate scenario for data interpretation, I recommend the authors include the data and, in addition to delta responses, conduct a multifactorial ANOVA and report main effects for the hemodynamic responses to capsaicin and tibial nerve stimulation.

Response: In the original version of our manuscript, baseline data for contraction and stretch

were given in figures 2 and 4, but was not given as text in the results section. We thought that giving baseline data in the figures was sufficient and that it would be redundant to restate this information as text. If the editor disagrees, we would be willing to repeat our baseline data as text in the results. In the original version, we omitted baseline data for the capsaicin experiments described in figure 1. This data has now been added to the figure. As requested, we have performed a one between-one within ANOVA on the responses to capsaicin and tibial nerve stimulation.

SENIOR EDITOR:

Comment: Referee 2, although generally satisfied with the responses to his first review, highlighted the need for a little more detail about the approach used to knock out the TRPV1 receptors without referring to the article cited by the authors. I suggest this is needed given the unique model.

Response: Please see below.

REFEREE COMMENTS

Referee #2:

Comment: I would like a little more specific detail about the approach used to knockout the TRPV1 receptors without having to refer to an article cited by the authors.

Response: As requested, we have added more information about the approach used to knockout TRPV1 receptors. In the original version of our manuscript, we stated that the CRISPR-Cas 9 method was used to delete 26 base pairs from exon 3 of the TRPV1 molecule. We have now added that the TRPV1 knockout rat was created by the Gene Editing Rat Resource Center at the Medical College of Wisconsin. More importantly, we also added that the knockout was generated by pronuclear injection of a CRISPR-Cas9 ribonucleoprotein targeting the sequence CTGCGATCATAGAGCCTCGG into 1 cell Sprague-Dawley rat embryos Stocker and Sullivan (2023).

Comment: It appears that numbers of animals reported for some of the analyses has changed from the first submission. The authors have not specifically highlighted these changes - or the reason(s) why - in the 'Responses to referees' document. This is a minor issue. Nevertheless, for transparency, I suggest that authors explain in their response why numbers differ between versions.

Response: The reason the numbers differ is that we performed a few additional experiments in order to verify the statistical significance of the findings.

Dear Dr Anselmi,

Re: JP-RP-2023-285267XR1 "Functional Knockout of the TRPV1 Channel Has No Effect on the Exercise Pressor Reflex in Rats." by Laura Anselmi, Guillaume P. Ducrocq, Victor Ruiz-Velasco, Sean D Stocker, Shannon P Higgins, and Marc P Kaufman

We are pleased to tell you that your paper has been accepted for publication in The Journal of Physiology.

TRANSPARENT PEER REVIEW POLICY: To improve the transparency of its peer review process, The Journal of Physiology publishes online as supporting information the peer review history of all articles accepted for publication. Readers will have access to decision letters, including Editors' comments and referee reports, for each version of the manuscript, as well as any author responses to peer review comments. Referees can decide whether or not they wish to be named on the peer review history document.

The last Word (or similar) version of the manuscript provided will be used by the Production Editor to prepare your proof. When this is ready you will receive an email containing a link to Wiley's Online Proofing System. The proof should be thoroughly checked and corrected as promptly as possible.

Authors should note that it is too late at this point to offer corrections prior to proofing. The accepted version will be published online, ahead of the copy edited and typeset version being made available. Major corrections at proof stage, such as changes to figures, will be referred to the Editors for approval before they can be incorporated. Only minor changes, such as to style and consistency, should be made at proof stage. Changes that need to be made after proof stage will usually require a formal correction notice.

All queries at proof stage should be sent to: TJP@wiley.com.

Are you on Twitter? Once your paper is online, why not share your achievement with your followers? Please tag The Journal (@jphysiol) in any tweets and we will share your accepted paper with our 30,000 followers!

Yours sincerely,

Harold D Schultz
Senior Editor
The Journal of Physiology
https://jp.msubmit.net
http://jp.physoc.org
The Physiological Society
Hodgkin Huxley House
30 Farringdon Lane
London, EC1R 3AW
UK
http://www.physoc.org
http://journals.physoc.org

P.S. - You can help your research get the attention it deserves! Check out Wiley's free Promotion Guide for best-practice recommendations for promoting your work at www.wileyauthors.com/eeo/guide. You can learn more about Wiley Editing Services which offers professional video, design, and writing services to create shareable video abstracts, infographics, conference posters, lay summaries, and research news stories for your research at www.wileyauthors.com/eeo/promotion.

IMPORTANT NOTICE ABOUT OPEN ACCESS: To assist authors whose funding agencies mandate public access to published research findings sooner than 12 months after publication, The Journal of Physiology allows authors to pay an Open Access (OA) fee to have their papers made freely available immediately on publication.

The Corresponding Author will receive an email from Wiley with details on how to register or log-in to Wiley Authors Services where you will be able to place an order.

You can check if your funder or institution has a Wiley Open Access Account here: https://authorservices.wiley.com/author-resources/Journal-Authors/licensing-and-open-access/open-access/author-compliance-tool.html.

EDITOR COMMENTS

Reviewing Editor:

I have no further comments.

Senior Editor:

Thank you for attending to the additional requests for revision. There are no more concerns. We are pleased to tell you that we accept your manuscript. Congratulations on a great study!

REFEREE COMMENTS

Referee #2:

Thank you for the additional revision and adding more detail as requested.

2nd Confidential Review **25-Sep-2023**
